# Mortalin and PINK1/Parkin‐Mediated Mitophagy Represent Ovarian Cancer‐Selective Targets for Drug Development

**DOI:** 10.1002/advs.202505592

**Published:** 2025-07-20

**Authors:** Vishal Chandra, Justin Garland, Rajani Rai, Donghua Zhao, Charuksha Walgama, Sadagopan Krishnan, Andrew T. Long, Tongzu Liu, Laura Adhikari, Doris M. Benbrook

**Affiliations:** ^1^ Gynecologic Oncology Section Stephenson Cancer Center Obstetrics and Gynecology Department College of Medicine University of Oklahoma Health Sciences Center Oklahoma City OK 73104 USA; ^2^ Pathology Department College of Medicine University of Oklahoma Health Sciences Center Oklahoma City OK 73104 USA; ^3^ Department of Physics Oklahoma State University Stillwater OK 74078 USA; ^4^ Department of Chemistry Oklahoma State University Stillwater OK 74078 USA; ^5^ Biochemistry Department College of Medicine University of Oklahoma Health Sciences Center Oklahoma City OK 73104 USA; ^6^ Present address: Department of Urology Zhongnan Hospital of Wuhan University Wuhan China; ^7^ Present address: Department of Physical & Applied Sciences University of Houston‐Clear Lake Houston TX 77058 USA

**Keywords:** autophagy, mitochondrial networking, mitophagy, PINK1, SHetA2

## Abstract

Mortalin is an essential chaperone for the import of nuclear‐encoded proteins into mitochondria and is elevated in ovarian cancer in association with poor patient prognosis. The investigational new drug, SHetA2, interacts with mortalin releasing its client proteins. In this study, interactions of SHetA2 moieties and mortalin substrate binding domain (SBD) amino acids are demonstrated by surface plasmon resonance (SPR) and nuclear magnetic resonance (NMR) to occur at low micromolar SHetA2 concentrations that selectively kill cancer cells over noncancerous cells. In both ovarian cancer and noncancerous cells SHetA2 reduces: mitochondria import of mortalin, degradation of mortalin's mitochondrial localization sequence (MLS), mortalin/inositol 1,4,5‐trisphosphate receptors complexes and oxidative phosphorylation. In cancer cells only, SHetA2 reduces calcium levels, mitochondrial length and fusion proteins, while inducing autophagy and PTEN‐induced kinase 1 (PINK1)/PARKIN‐mediated mitophagy. Noncancerous cells exhibit increased mitochondrial branch length in response to SHetA2 and a low level of inducible autophagy that is resistant to SHetA2. Inhibition of autophagosome‐lysosome fusion reduces, or increases, SHetA2 cytotoxicity in ovarian cancer or noncancerous cells, respectively. SHetA2 inhibits mortalin and growth, and induces mitophagy in ovarian cancer xenografts and increases survival post‐surgical tumor removal. In conclusion, SHetA2 binds directly to mortalin's SBD and causes distinct responses in ovarian cancer and noncancerous cells.

## Graphical abstract

Ovarian cancer patients with high levels of mortalin protein in their tumors have worse survival. The investigational drug SHetA2 interferes with mortalin's support of mitochondria. The resulting mitochondrial damage causes a process called mitophagy that contributes to how SHetA2 kills cancer cells. Noncancerous cells repair their mitochondria through fusion and are not killed by SHetA2.

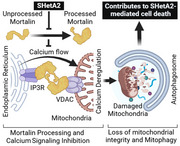

## 1 Introduction

Ovarian cancer has the highest ratio of mortality to incidence among all gynecological cancers.^[^ ^1^ ^]^ Owing to the lack of effective screening modalities, approximately 70% of patients are diagnosed at advanced stages of the disease, where it has already spread throughout the peritoneal cavity. Current treatment for advanced ovarian cancer includes a combination of platinum‐based chemotherapy and surgery. Despite initial chemosensitivity, recurrences are expected and occur within 18 months in 80% of patients diagnosed with advanced‐stage disease.^[^ ^2^ ^]^ The use of maintenance therapies, especially in patients with predicted biomarker‐expressing tumors, appears to have improved these statistics.^[^ ^3^, ^4^, ^5^ ^]^ Recurrences that occur more than 6 months following the initial treatment typically respond to retreatment with platinum‐based chemotherapy; however, the cancers often recur with decreasing time intervals between each recurrence.^[^ ^6^ ^]^ Patients with platinum‐resistant disease (recurrence <6 months from the last platinum therapy) have fewer treatment options and a survival estimate of <17 months.^[^ ^7^, ^8^ ^]^ Primary, maintenance, and second‐line therapeutics are clinically limited by the development of toxicities and recurrences in most patients.^[^ ^9^, ^10^, ^11^ ^]^ Based on these statistics, there is a clear need for novel drugs with improved efficacy and reduced side effects in patients with ovarian cancer.

Sulfur heteroarotinoid A2 (SHetA2) is a novel investigational new drug currently in clinical trial for the treatment of advanced cancer (clinicaltrial.gov: NCT04928508). This small molecule has been shown to prevent tumor establishment and growth without causing toxicity in multiple preclinical cancer models.^[^ ^12^ ^]^ Studies evaluating potential toxicity documented that SHetA2 did not cause skin irritancy, genotoxicity, or teratogenesis, and exhibited a no observed adverse effect level that was above 1500 mg kg^−1^ day^−1^, which is 50‐fold higher than the doses documented to inhibit cancer establishment and growth.^[^ ^13^, ^14^, ^15^, ^16^ ^]^ Three heat shock protein 70 kD (HSP70) proteins (mortalin, heat shock cognate 70, and glucose‐regulated protein 78) were implicated in the mechanism of SHetA2 by their isolation from ovarian cancer cell protein extracts using SHetA2 conjugated microspheres as bait.^[^ ^17^ ^]^ These proteins serve as molecular chaperones that promote folding of newly synthesized proteins, refolding or degradation of misfolded proteins, stability of protein complexes, and regulate degradation and intracellular localization of proteins.^[^ ^18^ ^]^ Specifically, mortalin is vital for mitochondrial function because it is essential for the import of nuclear‐encoded proteins into the mitochondria and refolds or promotes degradation of misfolded proteins within the mitochondria.^[^ ^19^, ^20^ ^]^

Mortalin is produced in a pre‐processed form with a mitochondrial localization sequence (MLS) that brings mortalin to mitochondria, where it is imported into the mitochondria through the mitochondrial import complex.^[^ ^21^ ^]^ This complex is a large multistructure machine that includes a “translocase of the outer membrane” (TOM) complex that recognizes MLS motifs on proteins and a “presequence translocase‐associated motor” (PAM) that drives the proteins through the “translocase of the inner membrane” (TIM) into the mitochondrial matrix.^[^ ^22^ ^]^ Mortalin plays multiple roles in this complex by assisting in bringing MLS‐containing proteins to TOM, hydrolyzing ATP to fuel PAM function and assisting in refolding of proteins as they enter through TIM.^[^ ^23^, ^24^, ^25^ ^]^ Because mortalin is the only PAM protein that contains an ATPase function, it is considered essential for the import of nuclear‐encoded proteins into mitochondria.^[^ ^20^, ^26^ ^]^ Inside the mitochondria, the MLS on mortalin and other proteins are proteolytically degraded, resulting in the processed form of mortalin or other proteins lacking an MLS.^[^ ^21^, ^27^ ^]^ Previously, only the processed form of mortalin has been detected in cells, suggesting that mortalin is imported into the mitochondria immediately after it is synthesized, and then, after its MLS has been removed it is translocated out of the mitochondria to other parts of the cell.^[^ ^21^ ^]^

Treatment of cancer cells with SHetA2 disrupts mortalin complexes with client proteins resulting in the client protein's degradation or altered localization within the cell, as well as mitochondrial damage.^[^ ^17^, ^28^, ^29^ ^]^ In cervical cancer cells, the mitochondrial damage was associated with mitochondria‐selective autophagy (mitophagy).^[^ ^30^ ^]^ Autophagy is a cellular recycling process in which double‐membraned autophagosomes form de novo in the cytoplasm, randomly surrounding molecules and organelles.^[^ ^31^ ^]^ Endpoints used to measure autophagy include microtubule‐associated protein light chain 3 (LC3) processing into LC3‐II and appearance of double‐membraned vesicles in electron micrograph images of cells.^[^ ^32^ ^]^ After formation, the autophagosomes fuse with lysosomes to form autophagolysosomes in which the contents are degraded by the acidity and proteases within. The resulting degradation products are then released to be used for the production of new molecules. There are two pathways by which autophagy becomes selective for mitochondria, the receptor‐mediated pathway in which BNIP3 and other proteins on the mitochondrial membrane bind to the LC3 during autophagosome formation, and another in which PINK1 is stabilized on the mitochondrial outer membrane, leading to the recruitment of PARKIN to the mitochondria.^[^ ^31^, ^33^ ^]^

Autophagy, mitophagy and other forms of selective‐mitophagy serve as survival functions by removing proteins and organelles damaged by age and various toxic stresses.^[^ ^34^ ^]^ When the various forms of autophagy occur in excess however, they can contribute to regulated cell death (RCD) by removing too many vital proteins and organelles before they can be replenished or by interacting with the RCD machinery.^[^ ^33^, ^34^ ^]^ Autophagy serves a cancer prevention role when it causes or contributes to the death of damaged cells before they become cancerous, and serves a cancer promotion role when it recycles damaged proteins and provides metabolic building blocks in nutrient‐poor conditions to support the heightened metabolism and survival of cancer cells.^[^ ^35^ ^]^

SHetA2 induces cell cycle arrest, mitochondrial damage, mitophagy, and apoptosis in cancer cells, while noncancerous cells are only sensitive to the cell cycle arrest effect. The reasons for the differential effect of SHetA2 on cancer compared to noncancerous cells are not known. Ovarian cancers express higher levels of mortalin in comparison to benign or borderline ovarian tumors,^[^ ^29^ ^]^ implicating mortalin in the mechanism of the differential SHetA2 activity. The objective of this study was to verify and characterize the binding of SHetA2 to mortalin and study the downstream effects of SHetA2 treatment in ovarian cancer and noncancerous cells from which some histologies of this cancer are known to originate, human ovarian surface epithelium (HOSE) and fallopian tube (FT) cells. Ultimately, the results could identify novel anticancer drug target candidates and/or support further study of already identified targets.

## 2 Results

### 2.1 Confirmation of SHetA2 Binding to Mortalin and Its Substrate Binding Domain (SBD)

**Figure 1:**
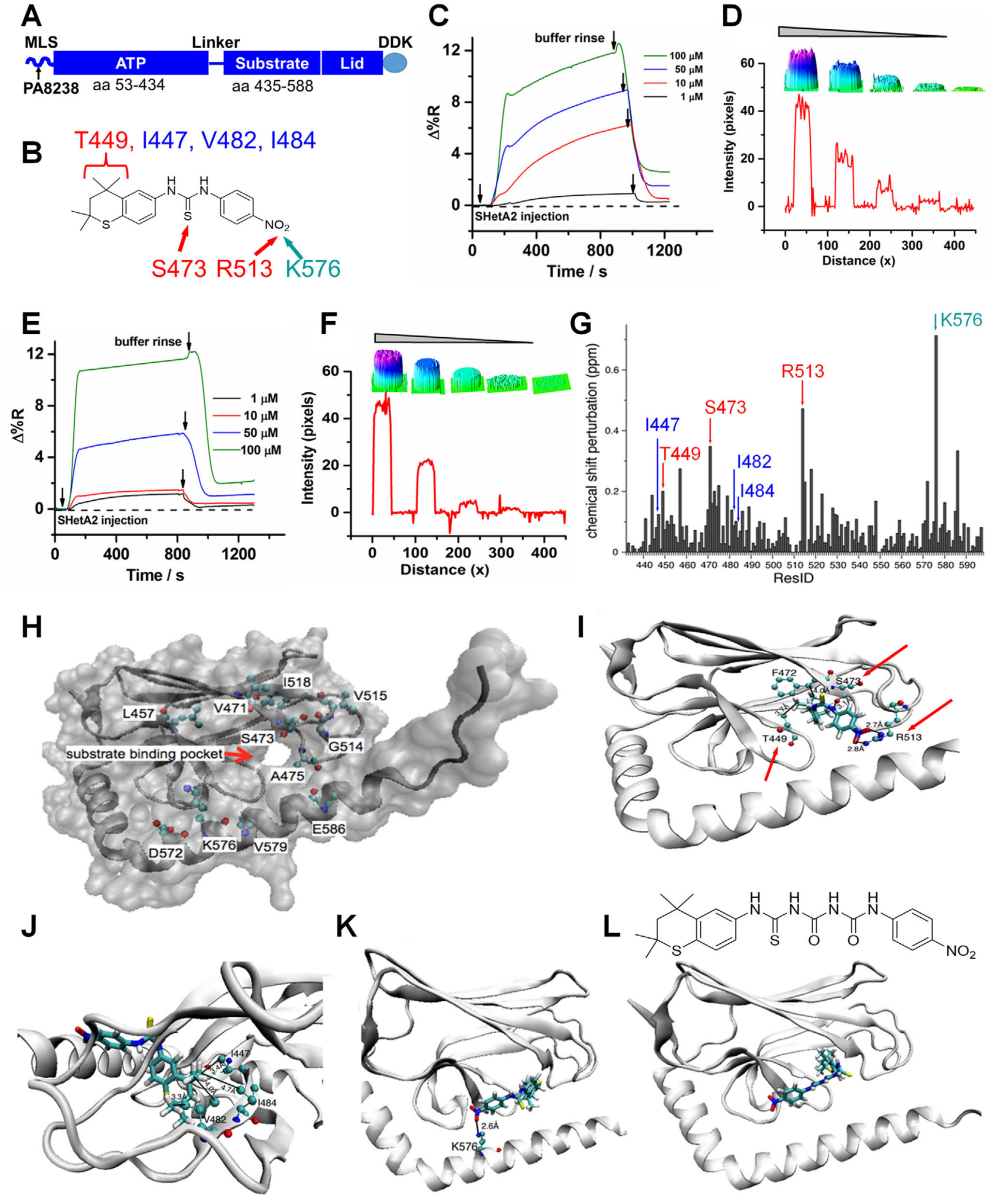
Identification of mortalin amino acids that interact with mortalin and confirmation of SHetA2 binding to mortalin substrate binding domain (SBD) (A). Mortalin protein structure with a DDK tag used in the NMR analysis. B) Chemical structure of SHetA2 and points of interaction with mortalin amino acids identified by NMR. The colored arrows and text refer to the amino acids identified to bind SHetA2 as shown in panels (G–K). C) Percentage reflectivity changes (∆%*R*), of the indicated concentrations of SHetA2 binding to surface immobilized full‐length mortalin. D) Line profile for the binding interactions in panel C (inset shows the 3D representations of the surface plasmon resonance (SPR) imager spots). E) ∆%*R* of SHetA2 with surface immobilized mortalin SBD. F) Line profile for the binding interactions shown in panel E (inset shows the 3D representations of the SPR imager spots). G) NMR chemical shift perturbation (difference between free and bound states),^[22]^ shows amino acid sites affected by the interaction with SHetA2; the program VMD is used for visualization.^[23]^ Binding of SHetA2 (H–K), and an analog (L), with the substrate binding domain of mortalin. **H**) NMR signals with over 0.2 ppm perturbation highlighted on the crystal structure and indicating specific mortalin SBD amino acids that interact with SHetA2. I) Two SHetA2 molecules docked onto the crystal structure of mortalin (PDB 3N8E), with the lowest energy −8.5 kcal mol^−1^. J) Another view of panel D showing additional hydrophobic interactions. K) Another docked configuration with −7.7 kcal mol^−1^ binding energy. L) Structure of SHetA2 analog with longer linker and its binding to mortalin.

To validate the interaction between SHetA2 and mortalin and identify the mortalin region involved, a surface plasmon resonance (SPR) microarray label‐free technique^[^ ^36^ ^]^ was used to measure SHetA2 binding to purified full length mortalin or the mortalin SBD in real time in vitro (**Table** **1**). The structural domains of the mortalin protein are shown in **Figure** **1**A and the chemical structure of SHetA2 is shown in Figure 1B.

**Table 1:** Kinetic binding parameters of SHetA2 to substrate binding domain (SBD) and full length mortalin.

Parameter	SBD	Full length
Association rate *k*_a_ (1/M*s)	150	101.5
Dissociation rate *k*_d_ (1/s)	0.0015	0.0003
Binding constant *K*_D_ (× 10^−6^ m)	10	3

The results demonstrate that SHetA2 can bind to full‐length mortalin (Figure 1C,D) and the mortalin SBD (Figure 1E,F) in concentration‐dependent manners. The calculated SPR imager‐based kinetic parameters (1–100 × 10^−6^ m) for SHetA2 binding to the full‐length mortalin are: association rate (*k*_a_) = 101.5 m ^−1^ s^−1^, dissociation rate (*k*_d_) = 3.0 × 10^−4^ s^−1^, and dissociation constant (*K*_D_) = 2.95 × 10^−6^ m. The kinetic parameters for SHetA2 binding to the mortalin SBD are: *k*_a_ = 150 m ^−1^ s^−1^, *k*_d_ = 1.5 × 10^−3^ s^−1^, and dissociation constant *K*_D_ = 10 × 10^−6^ m. The SHetA2 *K*_D_ for the SBD was over threefold greater than for the full length mortalin. These results document the physical binding of SHetA2 to the mortalin SBD.

### 2.2 Identification of Mortalin SBD Amino Acids that Interact with SHetA2

To identify the individual amino acids in the mortalin SBD that interact with SHetA2, nuclear magnetic resonance (NMR) experiments and molecular docking were performed (Figure 1G–L). The most perturbed signals with over 0.2 ppm chemical shift perturbation values^[^ ^37^ ^]^ were from L457, V471, S473, A475, G514, V515, I518, D572, K576, V579, and E586, which are highlighted on crystal structure of the same domain (PDB# 3N8E) in Figure 1H. Among this list, the first 7 surrounds the putative substrate binding pocket of the mortalin SBD (PDB# 1DKX of bacterial homolog DnaK in complex with a peptide) previously identified for peptide binding in another HSP70 protein DnaK.^[^ ^38^ ^]^ SHetA2 was then docked to mortalin with the large perturbation sites as additional inputs. In the configuration with the lowest binding energy of −8.5 kcal mol^−1^ (Figure 1I), interactions involve both amidine NH atoms in arginine (R513) with the two oxygen atoms of the nitro group in what appears to be a salt bridge. The hydroxyl group of serine (S473) forms an H‐bond with the nitrogen atom in the thiourea linker with a contact of 2.3 Å. A hydrophobic association occurs between one of the *gem*‐dimethyl groups of SHetA2 and the methyl group of threonine (T449). Additional hydrophobic interactions involve *gem*‐dimethyl groups and I447, V482, and I484 (Figure 1J). Figure 1K shows another low‐energy docked configuration (−7.7 kcal mol^−1^), highlighting the salt bridge between the lysine side chain amine group (K576) and the nitro oxygens. This is in good agreement with the very large chemical shift perturbation observed for K576 (Figure 1G). However, in this configuration, SHetA2 barely reaches the hydrophobic pocket. An SHetA2 analog designed with a longer flexible linker (Figure 1L) docks well to both the K576 site and the hydrophobic pocket as desired, exhibiting a lowered binding energy of −11.1 kcal mol^−1^. Therefore, these results indicate the existence of at least two binding sites. The NMR data roughly agree with the *K*_D_ = 1.0 and 3.7 × 10^−6^ m calculated from the SPR‐determined binding energies Δ*G* = −8.5 and −7.7 kcal mol^−1^ for the two lowest energy docking configurations, respectively.

### 2.3 Mortalin Processing and Mitochondrial Localization are Inhibited by SHetA2

**Figure 2:**
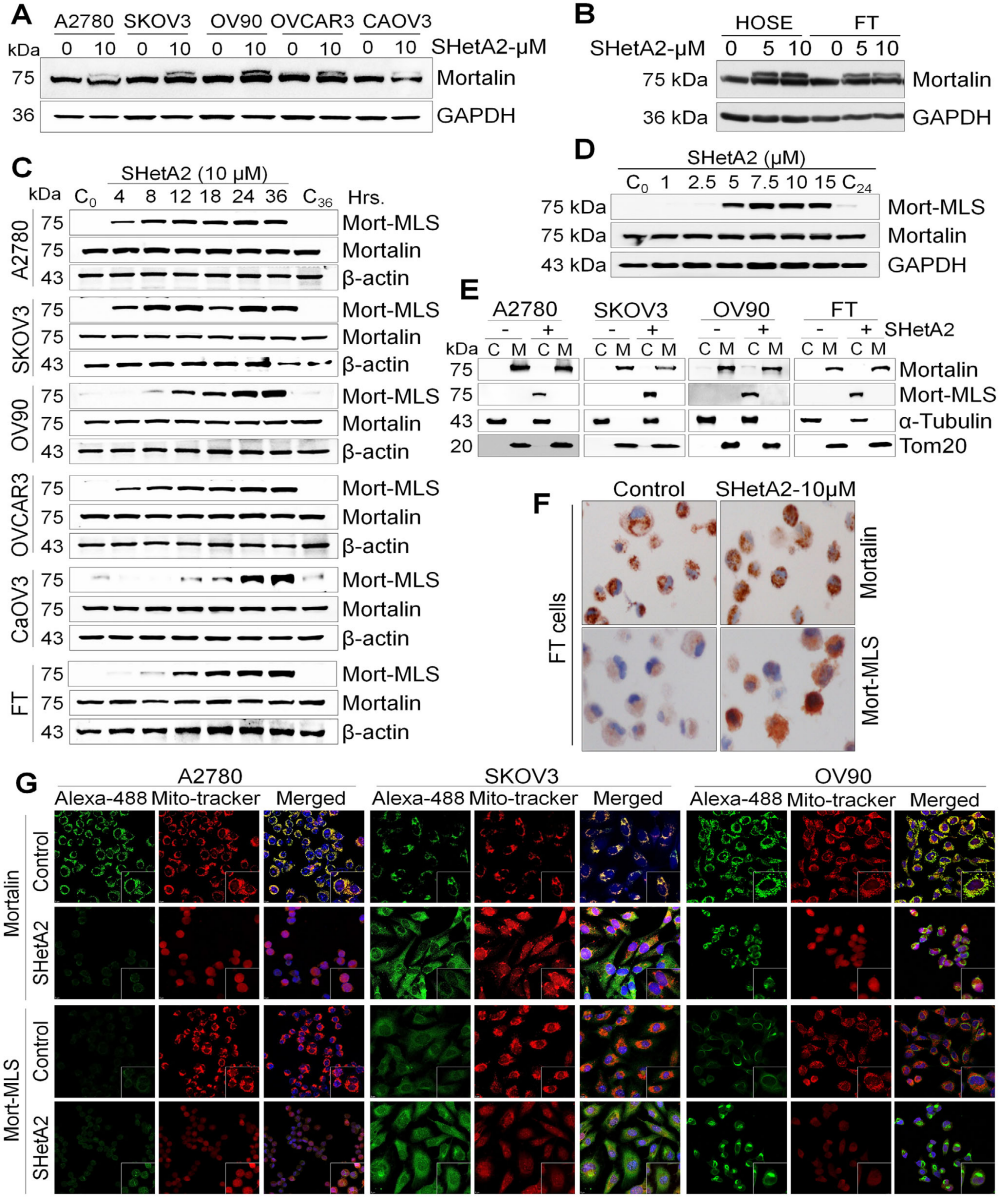
Mortalin processing and mitochondrial localization are inhibited by SHetA2. A,B) Ovarian cancer (A), and normal epithelial cells (human ovarian surface epithelium (HOSE) and fallopian tube (FT)), (B), were treated with dimethyl sulfoxide (DMSO), 5 and 10 × 10^−6^ m SHetA2 for 24 h, and protein isolates were immunoblotted for mortalin expression. C) Ovarian cancer cells and normal FT, cells were treated with DMSO or 10 × 10^−6^ m SHetA2 for various time‐points (indicated), and protein isolates were immunoblotted with mortalin and Mort‐MLS (which specifically binds to the MLS region of mortalin and recognizes only the unprocessed form) antibodies. D) SKOV3 ovarian cancer cells were treated with various doses (indicated) of SHetA2 for 24 h and protein isolates were immunoblotted for mortalin and Mort‐MLS antibodies. E) Ovarian cancer and FT cells were treated with DMSO or 10 × 10^−6^ m SHetA2 for 24 h and the subcellular fractions (C: Cytoplasmic and M: Mitochondrial) were immunoblotted with Mort‐MLS and mortalin antibodies. α‐Tubulin was used as cytoplasmic fraction marker, and Tom20 as a mitochondrial fraction marker. F) Immunofluorescence imaging was conducted with Mort‐MLS and mortalin antibodies in ovarian cancer cell lines treated with either DMSO or 10 × 10^−6^ m SHetA2 for 24 h. G) Imaging was performed using a confocal microscope at 63× magnification; Alexa‐488 (green) fluorescence was used to detect mortalin or Mort‐MLS, while MitoTracker (red) fluorescence was used to detect mitochondria. Yellow color indicates colocalization spots.

A major question arising from the findings that SHetA2 binds the mortalin SBD and disrupts mortalin/client protein interactions,^[^ ^17^, ^28^, ^30^ ^]^ is whether SHetA2 interferes with mortalin localization to the mitochondria. Because mortalin is required for mitochondrial import of nuclear‐encoded mitochondrial proteins,^[^ ^26^, ^39^ ^]^ SHetA2 could interfere with mortalin's ability to bring newly synthesized mortalin into mitochondria. The 46 aa MLS at the amino terminal of mortalin (Figure 1A) directs the precursor protein to the mitochondria, where the MLS is removed to form the processed form of mortalin, which is the predominant form of mortalin protein that has been detected in cells. Initial indications that SHetA2 affects mortalin import into the mitochondria were observed as the appearance of a lower mobility band in western blots of SHetA2‐treated ovarian cancer cell lines derived from multiple histologies and p53 statuses (**Figure** **2**A) and in noncancerous HOSE and FT cells (Figure 2B). This band was not present in lanes corresponding to control cultures treated with the same volume of vehicle (dimethyl sulfoxide/DMSO) used to administer the drug. The ability to detect this lower mobility band was dependent upon the commercial antibody used, with the anti‐Grp75/Mot mortalin antibody that recognizes both bands and the D13H4 mortalin antibody that recognizes only the higher mobility band. In all subsequent experiments, except for Figure 2F, we used D13H4 to evaluate the higher mobility in isolation from the lower mobility band. To determine whether the lower mobility band consists of the precursor form of mortalin with an intact MLS or a post‐translational modification of mortalin, such as phosphorylation, acetylation or sumoylation, we generated a rabbit polyclonal antibody to the mortalin MLS. This mortalin MLS antibody (Mort‐MLS) recognized only the lower mobility band in all cancer and noncancer cells treated with SHetA2 in time‐dependent (Figure 2C) and dose‐dependent (Figure 2D) manners, indicating that the lower mobility band consists of the unprocessed mortalin precursor and rules out the possibility that this band consists of other types of mortalin modifications.

To evaluate subcellular localization of mortalin, mitochondria‐ and cytoplasm‐enriched fractions were isolated from control and SHetA2‐treated cultures and evaluated by Western blot with the Mort‐MLS antibody and the commercial D13H4 antibody that recognizes only the total processed mortalin (higher mobility band), which confirmed that mortalin was primarily located in the mitochondria of untreated cells, while the mortalin MLS was only detectable in the cytoplasm of treated cells (Figure 2E). This same staining pattern was observed in cytochemical analysis of control and SHetA2‐treated FT cells using the Mort‐MLS and anti‐Grp75‐MOT mortalin antibodies (Figure 2F). Consistent with this finding, confocal imaging of cancer cell lines stained with fluorescent antibodies and organelle‐specific stains recognizing mortalin, mitochondria, and nuclei revealed that mortalin is present in a punctate pattern throughout the cells, which colocalizes with mitochondria in untreated cells, while SHetA2 treatment caused an increased and diffuse staining pattern for mortalin that did not colocalize with mitochondria (Figure 2G). These results demonstrate that mortalin processing and mitochondrial localization can be inhibited by SHetA2 in both cancer and healthy cells and that positive staining with the new Mort‐MLS antibody represents a candidate pharmacodynamic biomarker for SHetA2 exposure.

### 2.4 Mitochondrial Metabolism is Inhibited in Cancer and Noncancer Cells by SHetA2

**Figure 3:**
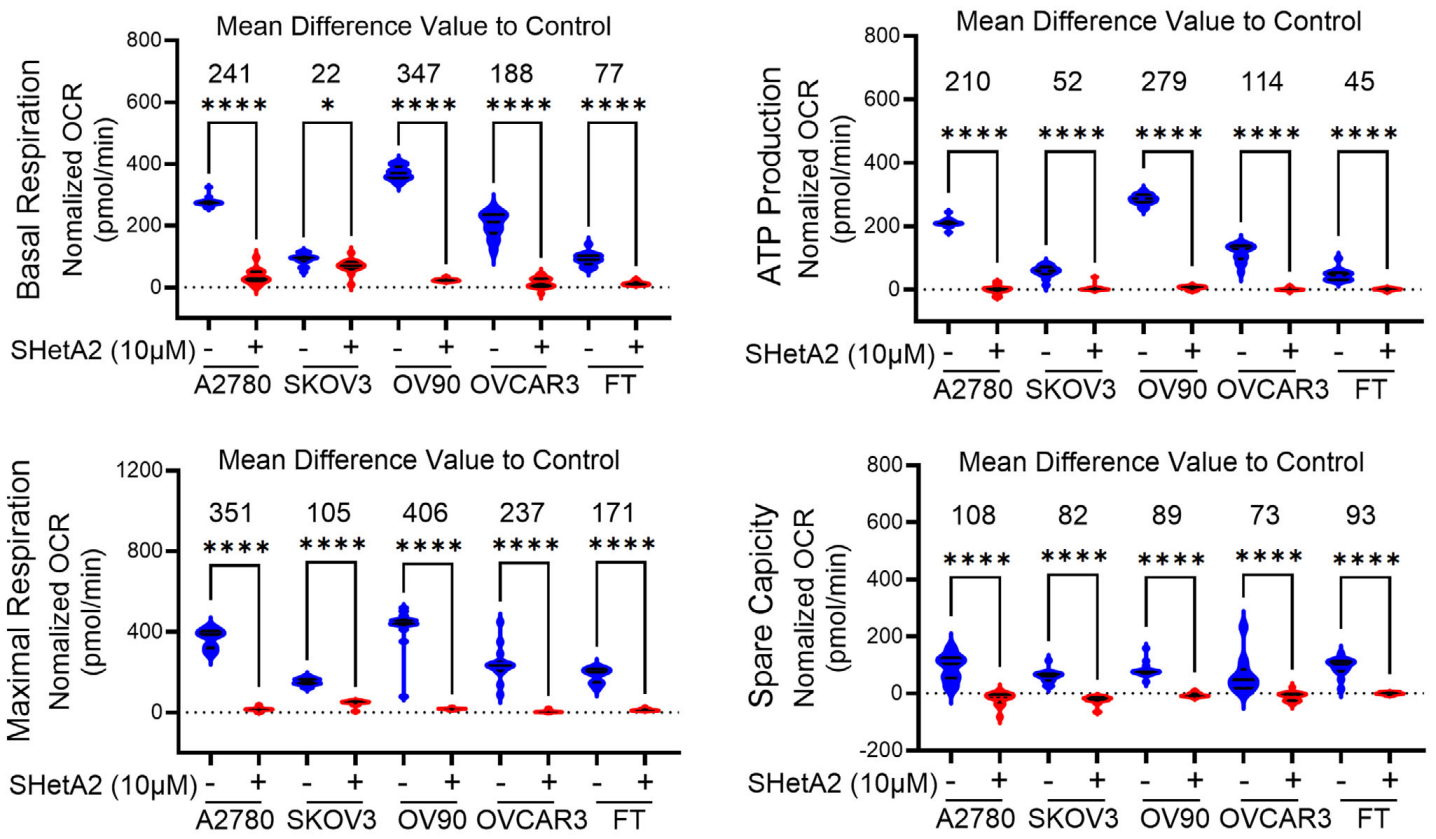
Mitochondrial metabolism is inhibited in cancer and noncancer cells by SHetA2. Normal or ovarian cancer cells were treated with DMSO or 10 × 10^−6^ m SHetA2 for 24 h and oxygen consumption rate (OCR) was measured using the Seahorse XFe96 analyzer and cell mito‐stress assay. Basal respiration (OCR, measured at starting point before injection of any substance representing the basic cellular ATP demand) ATP production (OCR measured after the injection of the ATP synthase inhibitor oligomycin representing ATP produced by the mitochondria that contributes to meeting the energetic needs of the cell) and maximal respiration (OCR measured after adding FCCP/uncoupler), spare respiratory capacity are shown. Data are shown as mean ± SD. “*p*” values are indicated ^*^ *p* ≤ 0.05, ^**^ *p* ≤ 0.01, ^***^ *p* ≤ 0.001, ^****^ *p* ≤ 0.0001 when compared with respective control.

While the critical role of mortalin in mitochondria biogenesis, function, maintenance, and response to stress is well established,^[^ ^20^ ^]^ less is known about the specific effects of inhibiting mortalin function on mitochondrial metabolism. The SHetA2 inhibition of mortalin mitochondrial localization was predicted to disrupt mitochondrial oxidative phosphorylation (OxPhos). Treatment with 10 × 10^−6^ m SHetA2 for 24 h blocked all aspects of oxidative phosphorylation in ovarian cancer cell lines or noncancerous FT cells (**Figure** **3**; Figure S1, Supporting Information).

### 2.5 SHetA2 Disrupts Mortalin/Inositol 1,4,5‐Trisphosphate Receptors Complexes and Mitochondrial Calcium (Ca^2+^) Levels in Ovarian Cancer, but Not in Noncancerous Cells

**Figure 4:**
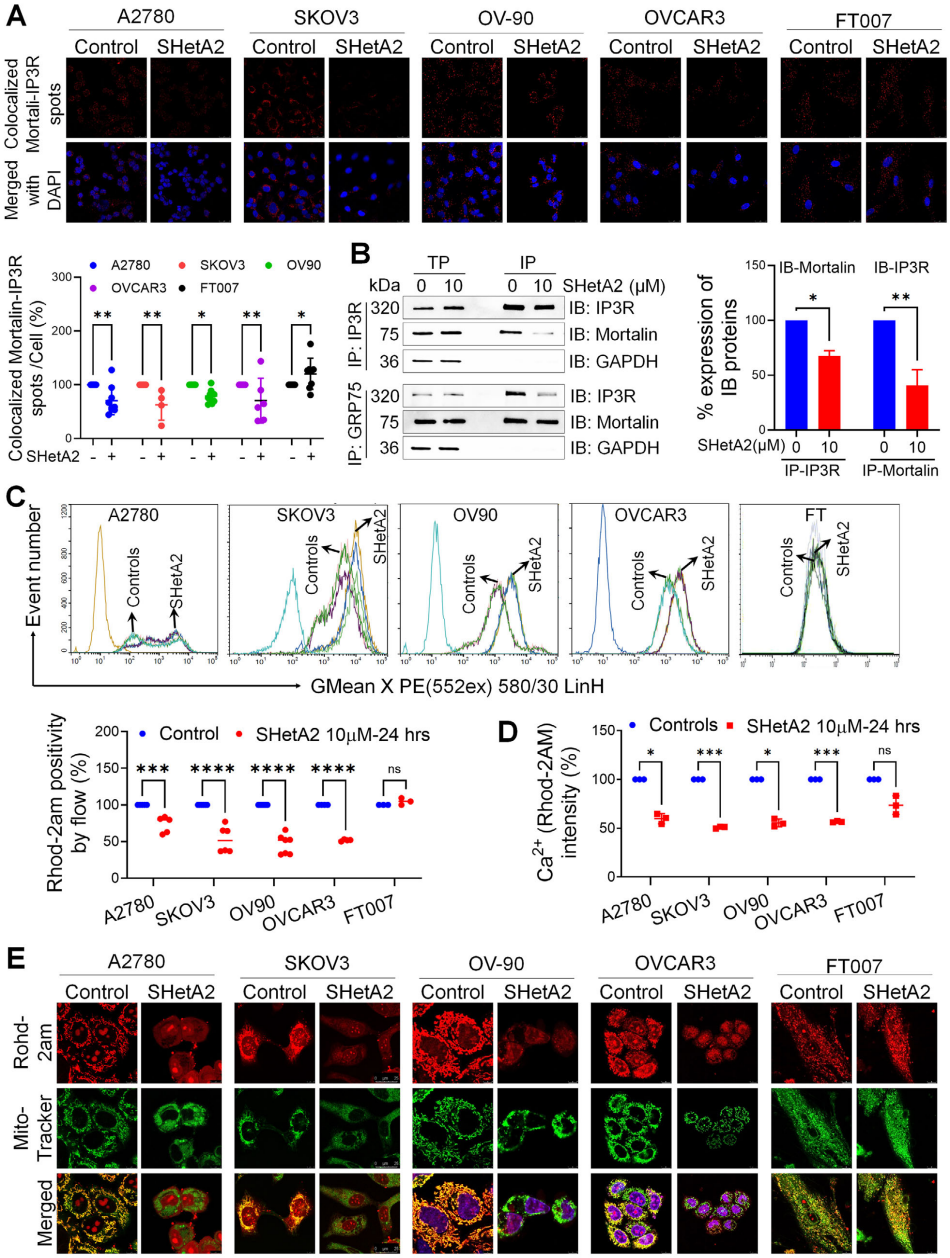
SHetA2 disrupts mortalin/inositol 1,4,5‐trisphosphate receptors complexes and mitochondrial Ca^2+^ levels in ovarian cancer, but not in noncancerous cells. A) Ovarian cancer and fallopian tube (FT) cells were treated with dimethyl sulfoxide (DMSO) vehicle or 10 × 10^−6^ m SHetA2 for 24 h and mortalin binding to IP3R was analyzed using proximity ligation assays (PLA), with mortalin and IP3R antibodies. Representative confocal images (63×) were shown. The PLA spots (of >6 images for each cell lines) were counted using the ImageJ software, normalized by the number of nuclei (DAPI stain, lower panel) and shown in the graph. B) OV90 ovarian cancer cells were treated with DMSO or 10 × 10^−6^ m SHetA2 for 24 h and collected protein isolates were coimmunoprecipitated (IP) with mortalin or IP3R antibody. Total protein and IP proteins were subjected to immunoblotting (IB, left panel). Densitometric analysis is shown in the bar graph (right panel). C,D) Ovarian cancer and FT cells treated with DMSO or 10 × 10^−6^ m SHetA2 for 24 h were stained with Rhod‐2‐AM and analyzed using flow cytometry (C), as well as BioTek plate reader at 552 nm (Ex), and 581 nm (Em) (D), to assess mitochondrial calcium levels. E) Ovarian cancer and FT cells treated with DMSO or 10 × 10^−6^ m SHetA2 for 24 h were stained with MitoView green and Rhod‐2AM to detect mitochondria and mitochondrial Ca^2+^ concentration, respectively and live cell confocal imaging was performed to detect mitochondrial localization of Ca^2+^. Confocal images were taken at excitation/emission wavelengths of Rhod‐2AM dye: 552 nm/581 nm and MitoView green: 490 nm/523 nm at a magnification of 63×. Yellow color indicates colocalization spots. Data are presented as mean ± SD. “p” values are indicated as ^*^ *p* ≤ 0.05, ^**^ *p* ≤ 0.01, ^***^ *p* ≤ 0.001, and ^****^ *p* ≤ 0.0001 when compared with the respective control.

The chaperone function of mortalin involves binding client proteins and assuring their proper folding and stability in complexes. We previously demonstrated that SHetA2 disrupts mortalin binding to p53 and p66shc.^[^ ^17^ ^]^ Mortalin binds to thousands of client proteins, however only a handful of proteins have been identified and validated as mortalin clients, one of them is the inositol 1,4,5‐trisphosphate receptors  (IP3R).^[^ ^28^ ^]^ Consistent with SHetA2 disruption of the mortalin/IP3R complex, proximity ligation assays (PLAs) demonstrated that SHetA2 reduced the numbers of positive puncta for mortalin/IP3 complexes in ovarian cancer cells but increased the number of positive puncta in noncancerous FT cells (**Figure** **4**A). Quantification of the puncta normalized to the total number of cells (identified as nuclei stained by DAPI) per image documented that SHetA2 reduced the positive puncta by 30, 37, 21, 29, and 20% in A2780, SKOV3, OV90, and OVCAR3, respectively, and increased positive puncta by 20% in FT cells. Validation of SHetA2 disruption of mortalin/IP3R complexes was done using the independent approach of coimmunoprecipitation of the proteins from SHetA2‐treated and untreated cultures. Coimmunoprecipitation with either an IP3R‐ or a mortalin‐specific antibody demonstrated that SHetA2 reduced mortalin/IP3R complexes in ovarian cancer cells by 32% or 59%, respectively (Figure 4B). Taken together with the SPR and NMR findings that SHetA2 binds inside the mortalin SBD, these results are consistent with SHetA2 binding to the SBD resulting in either prevention of IP3R binding to the mortalin SBD or release of IP3R from its binding site within the mortalin SBD.

Mortalin facilitates the transfer of Ca^2+^ from where it is released from endoplasmic reticulum (ER) through IP3R to the mitochondria through the mitochondrial calcium uniporter (MCU).^[^ ^40^ ^]^ Thus, SHetA2 disruption of mortalin/IP3R complexes was predicted to disrupt cellular Ca^2+^ dynamics. Consistent with this prediction, when compared to control cultures treated with DMSO vehicle alone, SHetA2 reduced total cell Ca^2+^ by 27, 47, 54, and 48% in A2780, SKOV3, OV90, and OVCAR3 cells, respectively, but had no effect on Ca^2+^ levels in healthy FT cells as measured by flow cytometry (Figure 4C) and plate reader (Figure 4D) measurements of Rhod‐2am fluorescence in intact cells. Also in comparison to control DMSO‐treated cultures, SHetA2 reduced Ca^2+^ levels specifically in mitochondria as demonstrated by confocal imaging of subcellular localization of Ca^2+^ in the mitochondria using MitoTracker Red to detect mitochondria and MitoView green to detect Ca^2+^ in the mitochondria (Figure 4E). Control DMSO‐treated cancer cells exhibited bright red (mitochondria) and green (Ca^2+^) puncta that colocalized, while SHetA2‐treated cells exhibited diffuse red and green staining that did not colocalize. In contrast, the punctate pattern and colocalization of red and green fluorescence staining was unchanged by SHetA2 treatment in noncancerous FT cells (Figure 4E). These findings demonstrate that SHetA2 selectively disrupts mitochondrial calcium homeostasis in ovarian cancer cells without affecting noncancerous FT cells.

### 2.6 Mitochondrial Structures are Altered Differentially in Cancer Compared to Noncancer Cells by SHetA2

**Figure 5:**
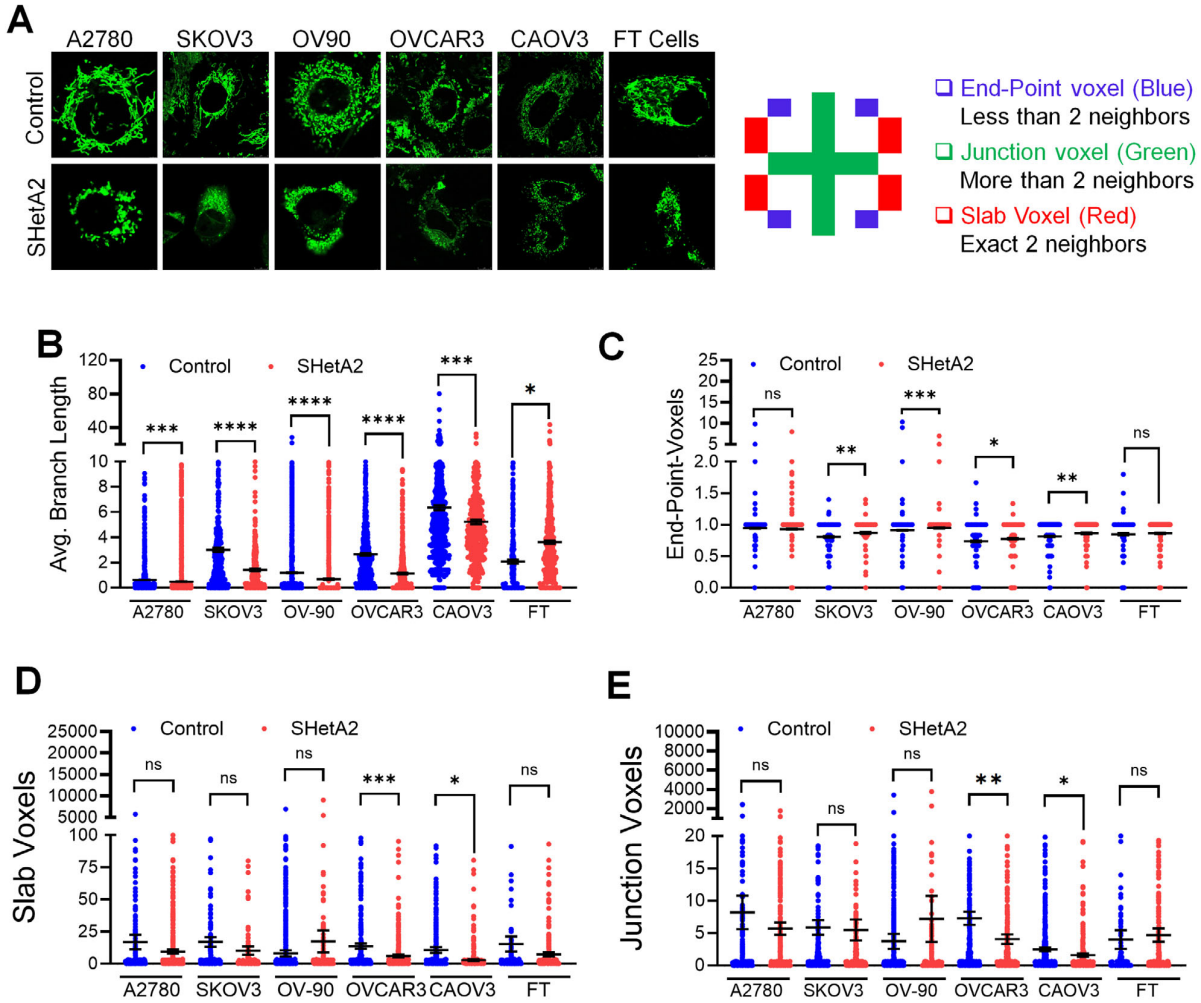
Mitochondrial structures are altered differentially in cancer compared to noncancer cells by SHetA2. A) Ovarian cancer and fallopian tube (FT) cells treated with dimethyl sulfoxide (DMSO) or 10 × 10^−6^ m SHetA2 for 24 h were stained with MitoView green and live cell confocal imaging was performed to detect mitochondrial networking. Confocal images were captured at 63× with 4× zoom to collect single cell image per capture (*n* = 10, A), and mitochondrial networks were analyzed using ImageJ software (B–E). Average mitochondrial branch lengths (B), end‐point voxels (C), slab voxel (D), and junction voxels (E), in DMSO versus SHetA2‐treated cells were compared by *t*‐tests. Data are presented as mean ± SD. “p” values are indicated as ^*^ *p* ≤ 0.05, ^**^ *p* ≤ 0.01, ^***^ *p* ≤ 0.001, and ^****^ *p* ≤ 0.0001 when compared with the respective control.

Consistent with SHetA2 disruption of mortalin function and mitochondrial Ca^2+^ levels observed, SHetA2 caused significant alteration in mitochondria structure in comparison to DMSO‐treated control cultures as observed with live cell confocal imaging using MitoView green to visual mitochondria in cells (**Figure** **5**A). SHetA2 caused decreased and increased average branch lengths in ovarian cancer cells and noncancerous FT cells, respectively (Figure 5B), endpoint voxels (representing absence or low numbers of branches) were significantly upregulated in four out of five SHetA2‐treated ovarian cancer cell lines, while no difference was observed in noncancerous FT cells (Figure 5C). Slab voxels (exactly two branches) and junction voxels (more than two branches) were also modulated by SHetA2 in an ovarian cancer cell line‐dependent manner, while normal FT cells remained unaffected (Figure 5D,E). These results suggest a dominance of mitochondrial fission over fusion (smaller, rounder mitochondria) in response to SHetA2 in cancer cells and the opposite (longer mitochondria) in noncancerous cells. To investigate this difference further, Western blot analysis was used to evaluate proteins involved in mitochondrial fusion (MFN‐1 and MFN‐2). The results demonstrated decreased MFN‐1 and MFN‐2 in SHetA2‐treated compared to control DMSO‐treated cultures of cancer but not noncancerous cells (Figure S2, Supporting Information). Taken together, these results suggest that cancer cells are defective in their ability to undergo mitochondrial fusion in response to SHetA2, while noncancer cells retain this capacity which may explain their relative resistance to SHetA2.

### 2.7 Cellular Response to SHetA2‐Induced Mitochondrial Damage and Mitochondria‐Selective Autophagy (Mitophagy)

**Figure 6:**
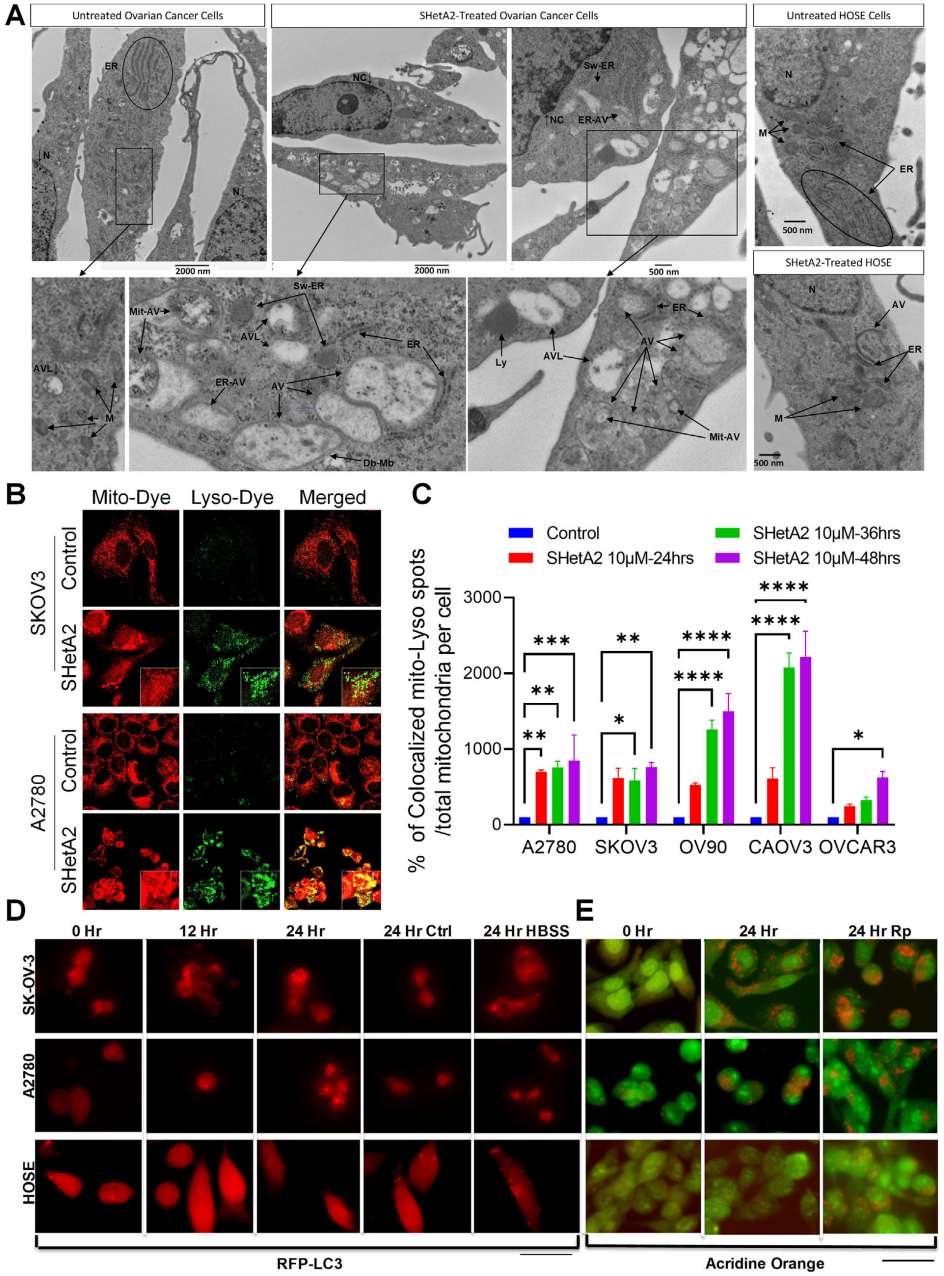
Cellular response to SHetA2‐induced mitochondrial damage and mitophagy. A) SKOV‐3 and human ovarian surface epithelium (HOSE) cells were treated with dimethyl sulfoxide (DMSO) or 10 × 10^−6^ m SHetA2 for 17 h and analyzed by electron microscopy to visualize endoplasmic reticulum (ER), mitochondria (M), nucleus (N), and autophagic vessels (AV). SHetA2 causes elevated levels of AV often surrounded by ER, ER inside of AV (Er‐AV), mitochondria inside of AV (Mit‐AV), autophagolysosomes (AVL), lysosomes (Ly), swollen ER (Sw‐ER), nuclear condensation (NC), and lack of M in cancer but not normal cells (A). B,C) Ovarian cancer cells treated with DMSO or 10 × 10^−6^ m SHetA2 for indicated time points were stained with Lyso‐Dye (green, recognizes lysosomes), and Mito‐Dye (red, recognizes mitochondria), and immunofluorescence imaging was performed to determine mitophagy. Representative confocal microscope at 63× were shown. Yellow color/colocalization spots indicates mitophagy induction (B). Mitophagy induction was quantified by dividing yellow spots by total mitochondria per cells and shown as bar graph (C). D) Normal or ovarian cancer cells were transfected with a GFP‐LC3‐tdTomato‐encoding plasmid, treated with SHetA2 (10 × 10^−6^ m), for the indicated time points, DMSO solvent (24 h Control), as a negative control, or serum starvation (6 h HBSS), a positive control and photographed at 40×. E) Normal or ovarian cancer cells were treated with SHetA2 (10 × 10^−6^ m), or Rapamycin (Rp), for 24 h and stained with acridine orange. Data are shown as mean ± SD. “*p*” values are indicated ^*^ *p* ≤ 0.05, ^**^ *p* ≤ 0.01, ^***^ *p* ≤ 0.001, ^****^ *p* ≤ 0.0001 when compared with respective control.

Electron micrographs of ovarian cancer cell SKOV3 and noncancerous HOSE cells treated for 17 h with 10 × 10^−6^ m SHetA2 or control solvent revealed that SHetA2‐induced mitochondrial swelling specifically in ovarian cancer cells, but not in healthy cells (**Figure** **6**A). In cancer cells, this treatment caused the cytoplasm to be almost filled with double‐membraned vesicles of a variety of densities reflecting autophagosomes and autophagolysosomes with a wide range of content degradation. Consistent with the ER as a source of de novo autophagic membrane formation in SHetA2‐treated cancer cells, normal and swollen ER were present surrounding the autophagosomes and ER was also observed inside the autophagosomes. Mitochondria in various states of degradation were present inside the autophagosomes while no mitochondria could be observed outside the autophagosomes. Treated cancer cells also exhibited nuclear condensation indicating that the process of apoptosis had begun, while nuclei of control cancer cells remained uncondensed. In comparison to the effects of SHetA2 observed on cancer cells, the noncancerous HOSE cultures appeared relatively unaffected. Basal levels of autophagic vesicles, mitochondria, ER structures and nuclei were apparent in both treated and control HOSE cells. While there was slight swelling of mitochondria in a subset of cells, the ER remained intact and the nuclei uncondensed.

To confirm that the SHetA2‐induced autophagy in cancer cells is selective for mitochondria (mitophagy), colocalization of mitochondria with lysosomes and involvement of specific mitophagy proteins were evaluated. A2780 and SKOV3 cells treated with SHetA2 exhibit a higher incidence of colocalized puncta for mitochondria and lysosome staining in comparison to control cells (Figure 6B). SHetA2 dose‐ and time‐dependent increases in mitochondria colocalization with lysosomes were observed in five ovarian cancer cell lines (Figure 6C; Figure S2, Supporting Information).

Molecular and live cellular assays were performed to confirm that mitophagy was induced in cancer, and not in noncancerous, cells upon SHetA2 treatment. Ovarian cancer and HOSE cells were transfected with a plasmid that expresses the LC3‐tdTomato hybrid protein. The tdTomato is a fluorescent peptide that allows the LC3‐tdTomato location to be tracked inside the cell with red fluorescent microscopy at 581 nm. The LC3‐tdTomato hybrid protein is localized diffusely throughout the cytoplasm and becomes punctate when incorporated into autophagic vesicles. The cancer, but not the noncancer, cells exhibited increased red puncta indicative of autophagic vesicles development with increasing time of SHetA2 treatment, or in response to serum starvation as a positive control (Figure 6C). Also, increased punctate acridine orange staining was observed in cancer, but not in noncancer, cells, treated with SHetA2 or rapamycin (positive control), indicating increased lysosomes or autophagolysosomes (Figure 6D). Low level autophagy was observed in the HOSE cells by the presence of a few puncta per cell, while inducible autophagy was observed upon serum starvation rapamycin treatment (Figure 6D,E). These results confirm SHetA2 selective induction of autophagy in cancer cells, while noncancerous cells exhibit a lower level of autophagy that is inducible by positive control treatments.

### 2.8 Molecular Mechanism of SHetA2‐Induced Mitophagy

**Figure 7:**
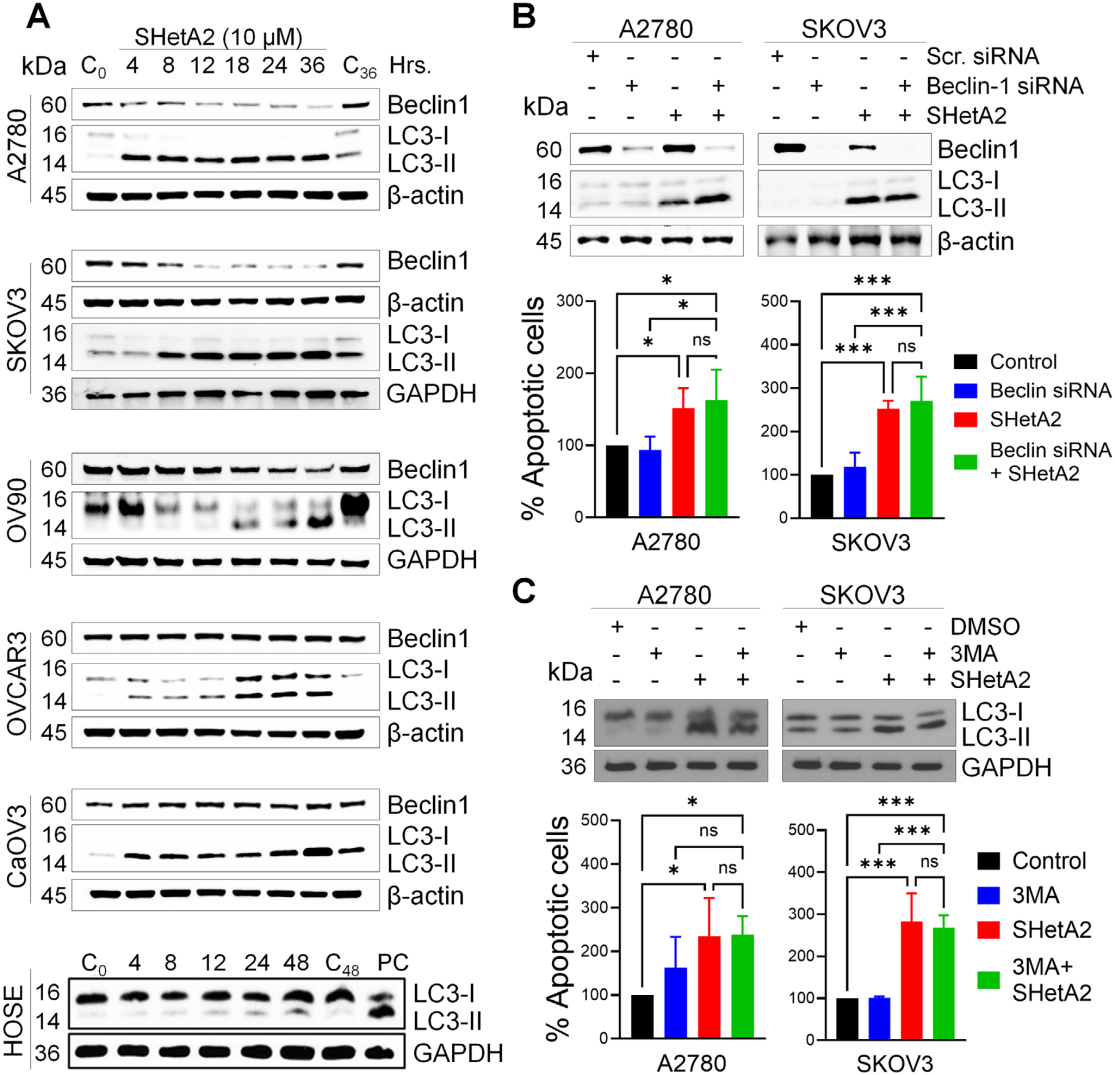
Molecular mechanism of SHetA2‐induced mitophagy. (A), Normal or ovarian cancer cells were treated with DMSO or 10 × 10^−6^ m SHetA2 for the indicated time points and protein isolates were immunoblotted to analyze the expression of Beclin1 and LC3‐I/II. β‐actin or GAPDH was used as loading control. B,C), Ovarian cancer cells treated by DMSO or 10 × 10^−6^ m SHetA2 for 24 h. in presence of initial autophagic inhibitors Beclin‐1 siRNA (B), and PI3K inhibitor‐3MA (C), and immunoblotting was performed to analyze the expression level of LC3‐I/II (upper panels), and for apoptosis induction by Annexin/PI assay (lower panels). Data are shown as mean ± SD. “*p*” values are indicated ^*^ *p* ≤ 0.05, ^***^ *p* ≤ 0.001, when compared with respective control.

At the molecular level, Western blot analysis confirmed time‐dependent elevation of LC3‐II in multiple ovarian cancer cell lines, while a much lower elevation was observed in noncancer HOSE cells (**Figure** **7**A). Beclin‐1 levels remained stable in OVCAR3 and CAOV3 cell lines but were decreased in A2780 and SKOV3 cell lines treated with SHetA2 over time. LC3‐II induction was evident as early as 4 h following SHetA2 treatment in all cancer cell lines, except in OV90 where it began after 18 h. Additionally, LC3‐II was detectable at the lowest tested concentration (1 × 10^−6^ m) of SHetA2 in A2780 and SKOV3 cell lines (Figure S3A, Supporting Information).

To determine if SHetA2‐induced mitophagy occurs through the classical autophagy initiation pathway, siRNA was used to test the role of Beclin‐1 and found to have no effect on the appearance of LC3‐II or apoptosis in SHetA2‐treated cancer cell lines (Figure 7B). Furthermore, inhibiting phosphoinositide 3‐kinase (PI3K) activity in the Beclin‐1 complex using 3‐methyl adenine (3‐MA) did not prevent LC3‐II induction in SHetA2‐treated cancer cell lines or apoptosis (Figure 7C). Overexpression of Bcl‐2 or Bcl‐xl to inhibit Beclin‐1 function^[^ ^41^ ^]^ similarly did not prevent SHetA2 induction of LC3‐II (Figure S3B, Supporting Information). Thus, the mechanism by which SHetA2 induces autophagy or mitophagy appears to be independent of Beclin‐1.

**Figure 8:**
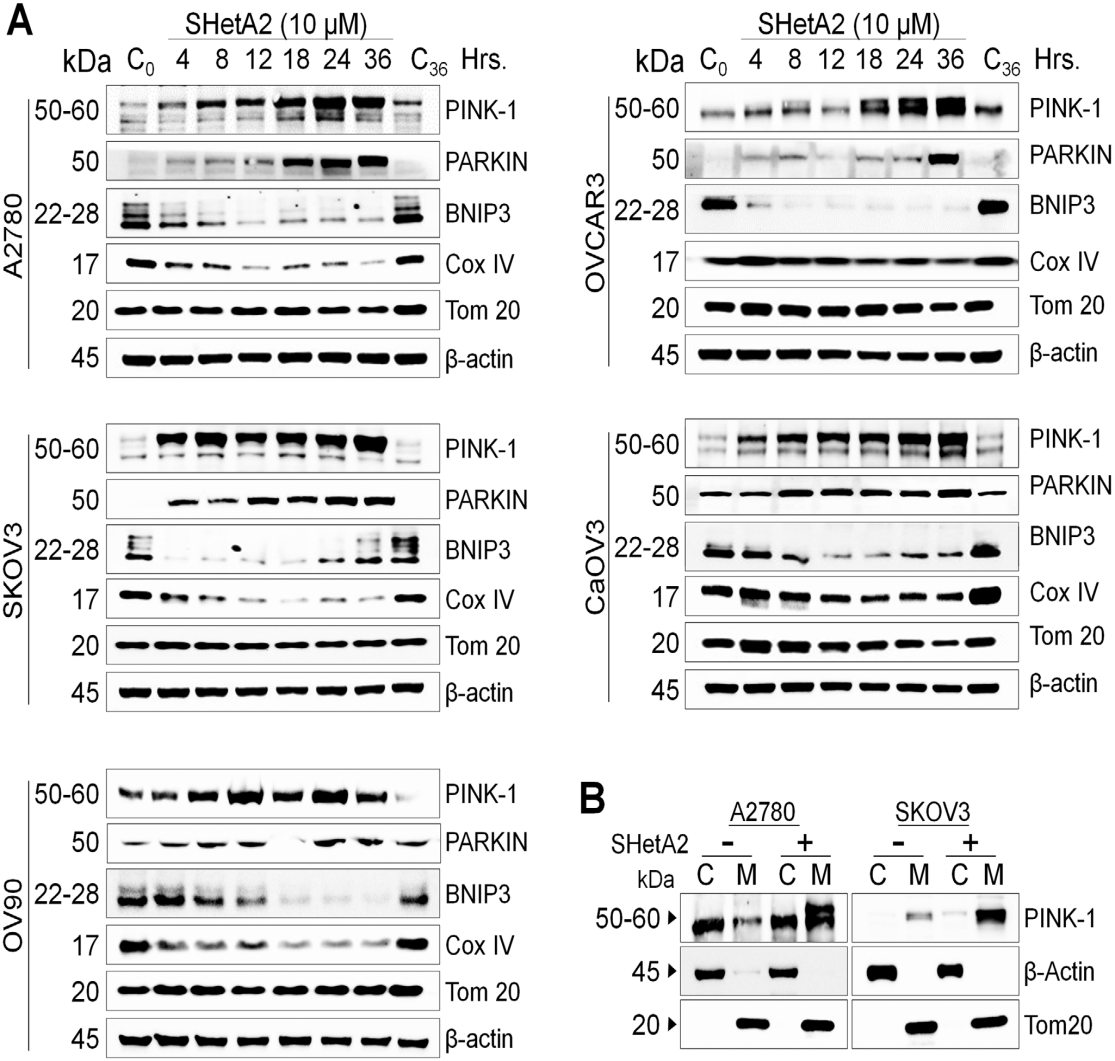
Molecular mechanism of SHetA2‐induced mitophagy. A) Ovarian cancer cell lines were treated with dimethyl sulfoxide (DMSO) or 10 × 10^−6^ m SHetA2 for the indicated time points and the protein isolates were analyzed for the expression of mitophagy markers including PINK‐1, PARKIN, BNIP3, and mitochondrial integrity markers including Cox IV and Tom 20 by immunoblotting. β‐actin was used as loading control. B) Ovarian cancer cells were treated DMSO or 10 × 10^−6^ m SHetA2 for 24 h and subcellular fractions (C: cytoplasmic and M: Mitochondrial), were collected and subjected to immunoblotting for PINK‐1 expression. β‐Actin was used as cytoplasmic fraction marker, while Tom 20 as a mitochondrial fraction marker.

To explore the mechanism of SHetA2‐induced mitophagy, its effects on multiple proteins known to be involved in mitophagy were evaluated by Western blot in cancer cells (**Figure** **8**A) and noncancerous FT cells (Figure S5, Supporting Information). The mitophagy receptor, Bcl2 interacting protein 3 (BNIP3)^[^ ^42^, ^43^ ^]^ exhibited inconsistent responses to SHetA2 treatment in cancer cells, but in general were at least temporarily reduced in comparison to control DMSO‐treated cancer cells. SHetA2 reduced mitochondrial‐localized cyclooxygenase‐IV (COX‐IV) protein in cancer cells, which protects cells from mitochondrial damage and apoptosis.^[^ ^44^ ^]^ The Tom20 protein, which is a member of the complex that mediates mitochondrial import of proteins^[^ ^45^ ^]^ was not affected by SHetA2 and therefore was used as an internal control for our mitochondrial subcellular fractionation. SHetA2 did not alter these proteins in comparison to the control DMSO vehicle control treatment in noncancer cells (Figure S5, Supporting Information).

Next, a Beclin‐1‐independent mechanism of mitophagy involving PINK1 and PARKIN^[^ ^46^ ^]^ was investigated in SHetA2‐treated cells. Western blot analysis demonstrated that SHetA2 caused time‐dependent increases in PINK1 and PARKIN levels in whole‐cell protein extracts (Figure 8A) and augmented the levels of PINK1 in mitochondria fractions with appearance of a lower mobility band suggestive of phosphorylation (Figure 8B).

### 2.9 Autophagy‐Lysosome Pathway Contributes to, or Interferes with, the Mechanism of SHetA2 Cytotoxicity in Cancer Cells or Noncancer Cells, Respectively

**Figure 9:**
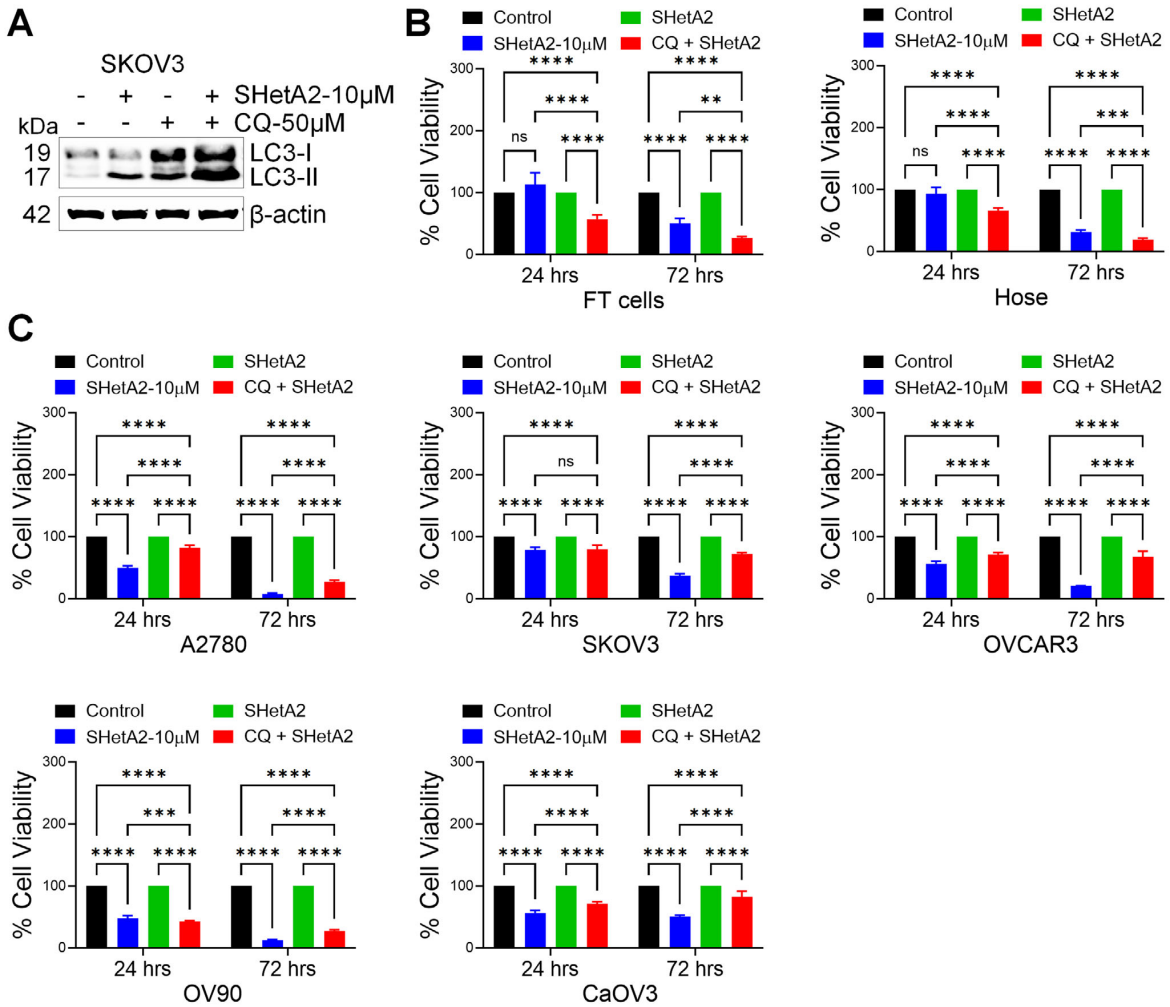
The autophagy‐lysosome pathway contributes to, or interferes with, the mechanism of SHetA2 cytotoxicity in cancer cells or noncancer cells, respectively. A) SKOV3 cells treated with dimethyl sulfoxide (DMSO), 50 × 10^−6^ m chloroquine (CQ), 10 × 10^−6^ m SHetA2 or their combination for 24 h and CQ mediated inhibition of autophagy was confirmed by immunoblotting to analyze the expression level of autophagy marker LC3 I/II. β‐actin was used as loading control. B) Noncancer FT and HOSE cells and C) Ovarian cancer cells were treated with SHetA2 in the presence or absence of CQ and cell viability was measured by MTT assay at indicated time points. “*p*” values are indicated ^*^ *p* ≤ 0.05, ^**^ *p* ≤ 0.01, ^***^ *p* ≤ 0.001, and ^****^ *p* ≤ 0.0001 when compared with respective control.

Inhibition of autophagosome‐lysosome fusion with chloroquine (CQ)^[^ ^47^ ^]^ caused elevation of LC3‐II in both the absence and presence of SHetA2 (**Figure** **9**A) consistent with accumulation of autophagosomes. Pretreatment with CQ reduced the efficacy of SHetA2 in cancer cells at both 24 and 72 h treatment times (Figure 9B). This suggests that autophagy or mitophagy serves as a mechanism of cell death in ovarian cancer cells. In contrast, CQ pretreatment reduced SHetA2 cytotoxicity in noncancer HOSE or FT cells (Figure 9C), indicating that autophagy functions as a mechanism of cell survival in this context.

### 2.10 Disruption of Mortalin Interactomes in Cancer and Noncancer Cells

To explore differences in the profiles of client proteins released from mortalin by SHetA2, coimmunoprecipitation experiments were performed on two ovarian cancer cell lines (OV90 and A2780) and two primary FT cultures (FT007 and FT032). Whole cell protein extracts of cells treated with SHetA2 or solvent were immunoprecipitated with the D13H4 mortalin‐specific antibody, and proteins released by SHetA2 from the precipitates were identified using mass spectrometry. All except one (H1F0) of the SHetA2‐released mortalin client proteins identified are known to be directly or indirectly involved in mitochondrial function, fission, fusion, and/or mitophagy (**Table** **2**). The PLEC protein was released from mortalin by SHetA2 in the two ovarian cancer cell lines. All other proteins released by SHetA2 were unique to each cell type.

**Table 2:** Proteins released from mortalin by SHetA2 treatment.

Protein	Cell type	Role in mitochondrial dynamics	Refs.
PLEC	OV90 and A2780	Mitochondrial fission followed by mitophagy is induced when PLEC anchoring of KRT8 to mitochondria is disrupted by KRT8 phosphorylation.	[48]
VCP	OV90	Inhibits mitochondrial fusion and promotes PINK1/PARKIN‐mediated mitophagy by degrading ubiquitinated proteins from the outer mitochondria membrane.	[49]
CLTC	A2780	Involved in formation of MitoPits on mitochondria membranes leading to mitochondria fission.	[50]
FASN	A2780	BNIP3‐mediated mitophagy stimulates FASN and cell survival under hypoxic conditions.	[51]
IQGAP1	A2780	II, Supports NDUSF4 of mitochondria complex I. IQGAP1 inhibition disrupts mitochondrial respiration leading to mitochondrial damage and mitophagy.	[52]
RPS15A	A2780	NI, involved in protein synthesis, cell cycle progression and repression of apoptosis.	[53]
RPLP2	A2780	II, involved in protein synthesis, its inhibition causes autophagy.	[54]
RPL13	OV90	Regulation of mitoribosome and OxPhos	[55]
ACTB	A2780	Actin cytoskeleton regulates mitochondrial dynamics and forms a cage around mitochondria during PINK1/PARKIN‐mediated mitophagy.	[56]
HBB	A2780	II, Supports oxygen levels in the inner mitochondrial membrane	
SNRPB	FT032	II, prognostically significant for patients with multiple myeloma when used with 4 other mitophagy‐associated genes in a risk model.	[57]
DDB1^a)^	FT007	Represses mitophagy through PINK1‐PARKIN and induces mitochondrial fission as complex with CLUF4A.	[58]

^a)^ Trend, adjusted *p* = 0.07, II = indirectly involved. NI = not involved.

### 2.11 Validation of the SHetA2 Mechanism in Animal Models

**Figure 10:**
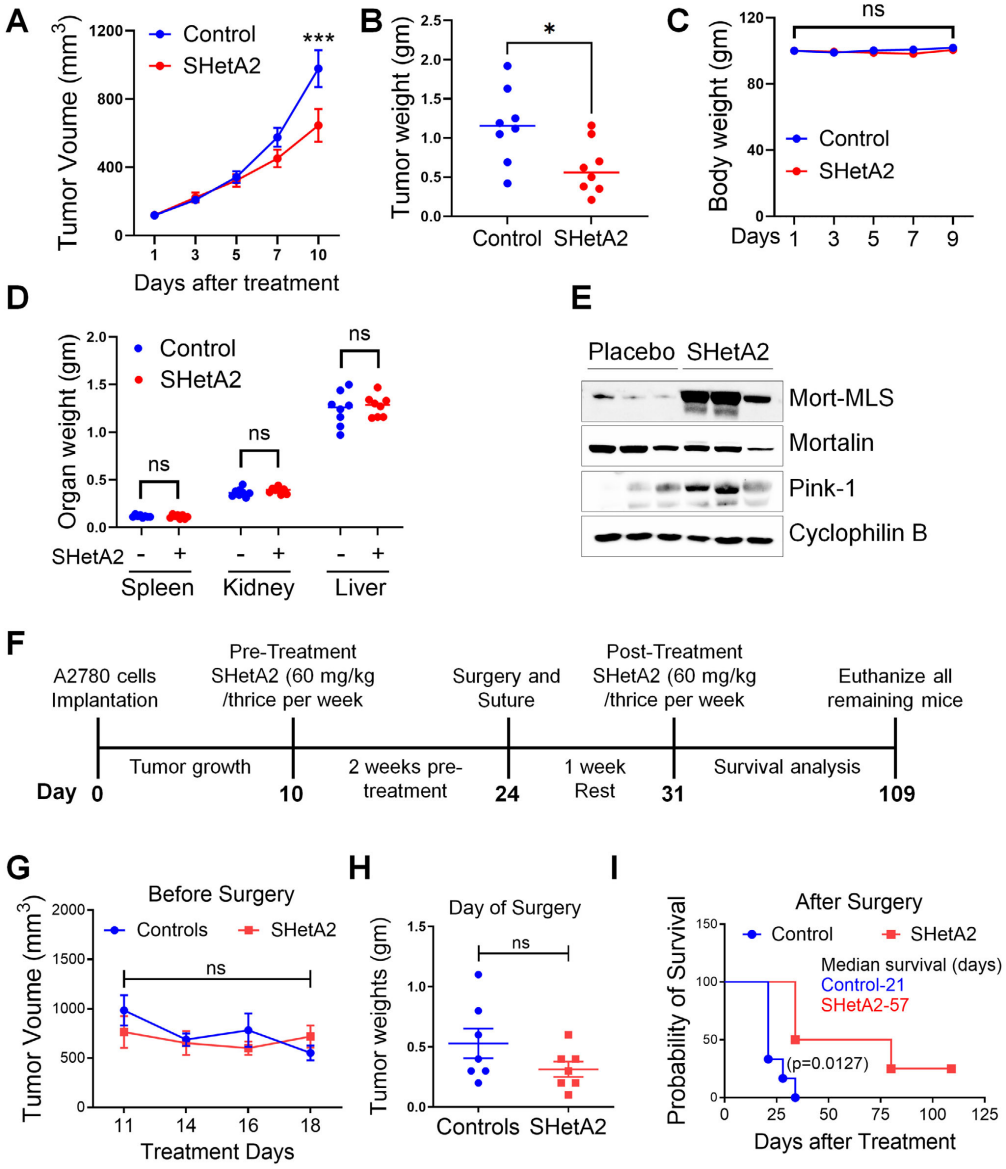
Validation of the SHetA2 mechanism in animal models. A–D) Effect of SHetA2 on average tumor volume (A), tumor weight (B), body weight (C), and organ weights (D), in OV90 ovarian cancer xenograft model (*N* = 8 mice). I, Protein isolates from tumor tissues obtained from the xenograft study were used for immunoblotting to detect the expression of mortalin MLS, Mortalin, and PINK‐1. F) The schematic representation of A2780 ovarian cancer xenograft study (*N* = 7 per group). G–I) Effect of SHetA2 on the average tumor volume (G), tumor weight (H), and survival (up to the 125 days) (I), of A2780 animal model. Unpaired student's *t*‐test was performed to evaluate the statistical difference. ^**^ *p* ≤ 0.01, ^***^ *p* ≤ 0.001 when compared with the respective control.

To validate the in vivo mechanism of SHetA2, we assessed its effects in an OV90 xenograft tumor model. SHetA2 was administered orally via daily gavage at a dose of 60 mg kg^−1^ to tumor‐bearing mice (*N* = 8 per group). Upon reaching a tumor diameter of 1000 mm^3^ in the control group, mice were euthanized, and their tumors were evaluated. SHetA2 induced a significant reduction in both tumor volume (**Figure** **10**A) and tumor weight (Figure 10B) by day 10 (necropsy day). To evaluate gross toxicity, body weight of the mice was monitored every other day throughout the study (Figure 10C), and spleen, kidney, and liver were collected and weighed at the end of the study (Figure 10D). Consistent with previous studies, SHetA2 did not significantly alter the body or organ‐to‐body weights of the mice. Mortalin MLS, mortalin, and PINK1 were evaluated in the tumors by Western blotting (Figure 10E) as pharmacodynamic endpoints for SHetA2.

In an alternative model, the effects of SHetA2 on animal survival following surgical removal of tumors was evaluated (Figure 10F). In this model, presurgical SHetA2 treatment was initiated when subcutaneous ovarian cancer xenograft tumors had appeared to reach their maximal size and stopped growing. A control volume of solvent (PEG400) or 60 mg kg^−1^ SHetA2 suspended in PEG400 was given to the mice by oral gavage three times per week. On the 10^th^ day of treatment, tumors were surgically excised by an experienced surgeon (T.L.) blinded to the treatment or control group assignments of the individual mice. SHetA2 or control solvent treatment were resumed 1 week later. Veterinarians blinded to the treatment group determined when each animal was beginning to suffer from their returning tumor burden, at which time the individual animal was euthanized. There were no significant differences between the control and treatment growths for tumor growth (Figure 10G) or size on the day of surgery (Figure 10H) (*N* = 7 animals each group, Wilcoxon Matched Pairs Signed Rank Test, *p* = 0.188). All of the animals in the control group died by day 34 post‐surgery, while 2 of the 7 animals in the SHetA2 treatment group were still alive on the last day (109) of the experiment (Figure 10I). The mean survival of the control and treatment groups were 21 and 57 days, respectively. The difference in survival between the control and treatment groups was statistically significant (Mantel‐Cox Log‐Rank Test, *p* = 0.011).

## 3 Discussion

The results of this study verify the physical binding of SHetA2 to mortalin and demonstrate that the downstream consequences of mitochondrial damage and mitophagy contribute to the mechanism by which this investigational new drug kills cancer cells without harming healthy cells. SPR analysis verified the physical binding of SHetA2 to full‐length mortalin and specifically the SBD of mortalin with kinetics that match the micromolar potency of SHetA2 for killing cancer cells.^[^ ^13^ ^]^ Independently, NMR analysis verified SHetA2 binding to the mortalin SBD and identified interaction between specific amino acids of the mortalin SBD and moieties of the SHetA2 chemical structure. The SHetA2 binding to the SBD instead of the ATPase domain of mortalin is consistent with the lack of SHetA2 toxicity because compounds that bind ATPase domains characteristically cause significant side effects due to their binding to ATPase domains of nontarget proteins.

Our study demonstrated that SHetA2 treatment excluded mortalin from the mitochondria and prevented proteolytic cleavage of the mortalin MLS. This supports the “chicken and egg paradox” concept that mortalin participation in the mitochondrial import complex is required for import of mortalin itself into the mitochondria. By interfering with the SBD of mortalin, SHetA2 can prevent mortalin from importing itself into mitochondria.

Similar to the consequences observed for genetic knockdown (KD) of mortalin,^[^ ^19^ ^]^ SHetA2 treatment of ovarian cancer cells caused mitochondrial damage, upregulation of PINK1 and PARKIN mitochondrial localization and Beclin‐1‐independent mitophagy. In the mortalin KD study, overexpression of PINK and PARKIN rescued SH‐SY5Y neuroblastoma cells that have mortalin KD. In our study, pharmacologic inhibition of mortalin with SHetA2 inhibited mitochondrial localization, mortalin MLS degradation and oxidative phosphorylation in both ovarian cancer and noncancerous FT or HOSE cells, while causing Ca^2+^ depletion, decreased mitochondrial branch length and autophagy/mitophagy only in the ovarian cancer cell lines. A similar pattern of differential SHetA2 effects has been documented by the demonstration of differential SHetA2‐induced RCD in multiple cancer types in comparison to noncancerous cells, and induction of cell cycle arrest in both cancer and noncancer cells.^[^ ^13^, ^59^, ^60^, ^61^, ^62^, ^63^ ^]^ This study begins to delineate the causes of SHetA2‐resistance in noncancerous cells.

An explanation for the reduced toxicity of SHetA2 to noncancerous cells in comparison to ovarian cancer cells could be that mitochondrial fusion capability and a low level of endogenous autophagy in noncancerous cells are sufficient to repair, and remove and recycle, respectively, the SHetA2‐damaged mitochondria to allow cell survival.^[^ ^64^ ^]^ In this study, SHetA2 decreased mitochondrial average branch length and voxels in ovarian cancer cells but increased mitochondrial average branch length and had no effect on voxels in noncancerous FT cells, supporting that noncancerous cells undergo mitochondrial fusion, a known survival mechanism. While SHetA2 had no effect on MFN‐1 and MFN2 in noncancerous cells, cancer cells exhibited decreased MFN‐1 and MFN‐2 proteins in response to SHetA2 treatment indicating that SHetA2 interferes with mitochondrial fusion in cancer but not in noncancerous cells. This differential SHetA2 decrease of MFN‐1 and MFN‐2 in cancer compared to noncancer cells makes cancer cells more vulnerable to SHetA2 perturbations of mitochondria, while noncancerous cells retain the survival mechanism of mitochondrial fusion. The low level of autophagy observed in noncancer cells in this study, is likely to be able to remove mitochondria that cannot be repaired by mitochondrial fusion. Consistent with autophagy playing a role in noncancer cell resistance to SHetA2, upregulation of autophagy was shown to be associated with resistance of noncancerous cells to inhibition of Ca^2+^ transfer from ER to mitochondria caused by inhibition of IP3R.^[^ ^65^ ^]^

The toxicity of SHetA2 to ovarian cancer cells was associated with the development of large mitochondria‐filled autophagic vesicles almost completely filling the cytoplasm. Inhibition of autophagosome‐lysosome fusion with CQ decreased the SHetA2 toxicity in cancer cells, but and increased SHetA2 toxicity in healthy cells, indicating that SHetA2 causes mitophagy‐related cell death selectively in the ovarian cancer cells, while mitophagy serves a protective role from SHetA2 in noncancerous cells. Mitophagy plays regulatory roles in multiple different forms of RCD.^[^ ^33^ ^]^ While inhibition of mitophagy has been shown to promote apoptosis and increase sensitivity of breast cancer cells treated with chemotherapeutic agents,^[^ ^66^, ^67^ ^]^ it has been shown to contribute to drug‐induced pyroptosis in glioma cells.^[^ ^68^, ^69^ ^]^ Similar to the SHetA2 mechanism revealed in this study, cancer‐selective induction of mitophagy contributed to the mechanism of preferential cytotoxicity to cancer cells by sodium selenite.^[^ ^69^ ^]^ Other studies supporting a role for mitophagy in cancer drug resistance demonstrated that cannabidiol, melatonin and sorafenib increase drug sensitivity of glioblastoma and hepatocellular carcinoma cells by inducing mitophagy.^[^ ^33^ ^]^ SHetA2‐induced intrinsic apoptosis in multiple cancer types including ovarian, cervical, endometrial, kidney and head and neck squamous cancer,^[^ ^28^, ^30^, ^59^, ^60^, ^70^ ^]^ and sensitized ovarian and lung cancer cells to extrinsic apoptosis,^[^ ^71^, ^72^, ^73^ ^]^ however it is possible that this drug also induces other forms of RCD depending on the context. This study and an evaluation of the mechanism of SHetA2 in cervical cancer cell lines^[^ ^30^ ^]^ support that the mechanism of SHetA2‐induced cell death in these two cancer types involves mitophagy‐related RCD. More research on SHetA2‐induction of RCD is warranted to optimize the translation of this drug to later phase clinical trials.

Another explanation for the differential SHetA2 sensitivity of cancer compared to noncancerous cells is that the cancer cells have become addicted to elevated levels of mortalin. We and others have shown that mortalin expression is increased during ovarian cancer development and is associated with prognosis of patients with ovarian cancer.^[^ ^29^, ^74^, ^75^ ^]^ Elevation of mortalin during carcinogenesis could help to maintain the function of mitochondria that are stressed by increased demand for metabolic products.

Because many drugs fail in early stage clinical trials, it is important to optimize the clinical trial design by identifying those patients most likely to benefit from the treatment. Based on the results of this study a biomarker of mitophagy capacity or sensitivity, such as basal or inducible PINK and PARKIN, could be considered for identifying patients with tumors that could be pushed into mitophagy‐related RCD. Monitoring whether experimental drug exposure is sufficient to reach the tumor and molecular targets is another important aspect of optimizing clinical trial design. The Mort‐MLS antibody developed in this study to detect the mortalin MLS could be used to measure unprocessed mortalin as a pharmacodynamic endpoint. Our mouse models document that SHetA2 inhibition of xenograft tumor growth is associated with alterations in mortalin and PINK‐1, and that pre‐ and post‐surgical treatment with SHetA2 can increase overall survival. Another promising pharmacodynamic endpoint to monitor SHetA2 exposure in clinicals trials that has been validated in in vivo models is degradation of cyclin D1.^[^ ^59^, ^76^, ^77^, ^78^, ^79^, ^80^, ^81^, ^82^ ^]^

Because greater knowledge of the SHetA2 molecular mechanism is needed to optimize clinical trial design, we further explored the mortalin binding proteins disrupted by SHetA2 in various cell types. In a hypothesis‐driven approach we demonstrated that SHetA2 disrupts mortalin IP3R interaction, which is critical for maintenance of mitochondrial Ca^2+^ and metabolism. Although mortalin has many client proteins,^[^ ^83^ ^]^ our exploratory mass spectrometry approach identified only one or a few client proteins known to be released from mortalin by SHetA2 in each of the cell types studied. Validation of the integrity of this approach used was provided by the identification of VCP being released from mortalin by SHetA2 in both ovarian cancer cell lines, and not in either of the noncancerous cell cultures, evaluated. Furthermore 10 of the 11 proteins identified to be disrupted from mortalin by SHetA2 have direct or indirect roles in mitochondrial dynamics, which is consistent with the essential function that mortalin plays in maintaining mitochondrial integrity. The majority of these proteins are involved in the PINK1/PARKIN pathway of mitophagy shown to be induced by SHetA2 in this study. SHetA2 release of client proteins from mortalin can lead to either degradation of the client protein or its relocation within the cell, leading to either suppression or activation of their cellular functions.^[^ ^29^, ^59^, ^82^ ^]^ Thus, investigation of each of these identified proteins is needed to fully elucidate their mechanistic involvement in SHetA2‐mediated mitophagy.

In summary, this study demonstrates that interference with mortalin can selectively induce PINK1/PARKIN‐mitophagy‐related RCD in ovarian cancer compared to noncancerous cells. SHetA2 exerts this activity through a novel mechanism involving binding to the mortalin SBD and inhibition of mortalin mitochondrial import. The equal sensitivity of mortalin and differential sensitivity of mitochondria to SHetA2 treatment in ovarian cancer compared to noncancerous cells suggests that cancer cells have become addicted to elevated mortalin and that noncancerous cells can resist SHetA2 treatment through mitochondrial fusion and autophagy.

## 4 Experimental Section

### Reagents, Cell Lines, and Cultures

SHetA2 was synthesized by K. Darrell Berlin, PhD (Oklahoma State University, Stillwater, OK, USA) according to published methods.^[^ ^63^ ^]^ After synthesis the solvent was evaporated and SHetA2 was recrystallized to produce an analytically pure sample as validated by HPLC analysis. Ovarian cancer cells lines A2780 (RRID: CVCL_0134), OV‐90 (RRID: CVCL_3768), SKOV3 (RRID: CVCL_0532), CAOV3 (RRID: CVCL_0201), and OVCAR3 (RRID: CVCL_0465) were purchased from American Type Culture Collection/ATCC (Manassas, VA, USA). HOSE cell line was a gift from Dr. Danny N. Dhanasekaran (University of Oklahoma Health Sciences Center, Oklahoma City, OK, USA). The pBL‐GFP‐LC3‐tdTomato plasmid was a gift of Dr. Guangpu Li (University of Oklahoma Health Sciences Center, Oklahoma City, OK, USA) who generated the construct by cloning the tdTomato‐LC3‐eGFP cassette into the Mlu/Nhe site of the pBI plasmid (Kusatsu, Shiga, Japan). The pCDH‐CMV‐MCS‐EF1‐Puro‐BCL2 and 3120 pSFFV‐neo BCL‐XL plasmids were purchased from Addgene (Watertown, MA, USA). Human fallopian tube secretory epithelial cells (FT cells) were isolated from fallopian tube fimbriae surgical specimens under an IRB approved protocol as previously described.^[^ ^84^ ^]^ All cell lines were authenticated by autosomal short tandem repeat (STR) profiles determination within 3 years and comparison with reference databases by the core facility. M‐PER Mammalian Protein Extraction Reagent (#78501), T‐PER tissue protein extraction reagent (#78510), BCA reagent (#23227), Apoptosis assay kit Annexin V/Dead Cell apoptosis kit (#V13241), MitoTracker Red CMXRos (#M7512), Prolong Gold mounting medium with DAPI (#36962), Pierce Co‐Immunoprecipitation Kit (#26149), Rhod‐2AM (#R1245MP) were purchased from Thermo Fisher Scientific (Waltham, MA, USA). Mitochondria/Cytosol Fractionation Kit (ab65320), secondary antibody tagged with fluorochrome, Alexa Fluor 488 (#ab150077), 647 (#ab150115) and the mortalin Anti‐Grp75/MOT antibody (#ab53098) were purchased from AbCam (Cambridge, MA, USA). Polyclonal Mort‐MLS, antibody was synthesized from Thermo Fisher. Antibodies, anti‐Pink1 (#6946), ‐PARKIN (#4211), ‐Cox‐IV (#4850), ‐BNIP3 (#3769), ‐TOM20 (#42406S), ‐p62 (#5114s), ‐αTibulin (#2125S), ‐BCL‐XL (2764S#), ‐BCL2 (4223S#), ‐LC3 I/II (#12741S), ‐Beclin‐1 (#3738s), ‐DDK (#14793s), ‐IP3R (#8568) and ‐Mortalin Grp75 (D13H4)‐XP (#3593s), anti‐GAPDH (#5174), anti‐β‐actin (#4970), secondary antibodies conjugated with horse radish peroxidase (HRP), anti‐mouse (#7076S) or anti‐Rabbit (#7074V), Scrambled or Beclin1 siRNA (#6222) were purchased from cell signaling technology (Danvers, MA, USA). Duolink PLA Probes and PLA Fluorescence in situ Detection Kit Red (#DUO92004, #DUO92002, #DUO92008), Rapamycin (#R0395), Autophagy inhibitor‐3MA (#M9281), Acridine orange (#318337), CQ (#C6628), Hoechst (#H3570) were purchased from Sigma (St Louis, MO, USA). Cell Titer 96 Non‐Radioactive Cell Proliferation Assay (MTT) (#G4100) was purchased from Promega (Madison, WI, USA). Transfecting reagent HiPerfect (#301704) and lipofectamine 3000 reagent (#L3000008) were purchased form Qiagen (Hilden, Germany) and Thermo Fisher Technology respectively. Chamber slides (#154526) were purchased from Nunc Lab‐Tek II Chamber Slide System from Thermo Fisher. Black clear bottom Cell carrier^TM^ ‐96 well plate (#NC1463153) were purchased from Perkin Elmer (Waltham, MA, USA), Mitophagy Detection Kit (#MD01‐10) was purchased from Dojindo Molecular Technologies (Rockville, MD, USA), XF Cell Mito Stress Test Kit (#103708‐100) were purchased from Agilent Technologies (Santa Clara, CA, USA), MitoView Green (#70054), was purchased from Biotium (Fremont, CA, USA).

### Protein Purification for SPR and NMR Experiments

Plasmid pET‐52 encoded with mortalin SBD (residues 439 to 597) gene, the full‐length wild type (WT) mortalin gene with the MLS deleted, or a truncation containing only the mortalin SBD were transformed into *E. coli* BL21 (DE3) cells, which were grown with M9 minimal medium enriched with ^13^C‐glucose and ^15^NHCl_4_ as the only carbon and nitrogen sources. The cell culture was incubated at 37 °C until OD600 reached 0.8 and then incubated at 20 °C for 20 h with 1 × 10^−6^ m Isopropyl‐β‐D‐thiogalactoside for induction. Then cells were harvested by centrifugation and lysed by incubation with lysozyme at room temperature followed by sonication. The protein was found in the soluble portion. The lysates were forced through a 0.2‐micron filter to remove remaining cell debris and then transferred to a Ni‐NTA IMAC column for protein purification. The column was washed with 10 × 10^−3^ m imidazole buffer and eluted with 1 m imidazole. The eluted fractions were buffer exchanged to 20 × 10^−3^ m Tris‐buffer (pH 7.4) and subsequently digested by HRV‐3C protease to remove His‐tag. The cleaved product was passed through Ni‐NTA column again to remove the cleaved His‐tag. The purity of the proteins were determined to be >95% by SDS‐PAGE. The purified protein was concentrated to 20 mg mL^−1^ and stored at 4 °C before use.

### SPR Binding Assay

An SPR microarray imaging method was conducted using a GWC SPRimager‐II connected to a Pico Plus Elite Pump11 (Harvard Apparatus, MA, USA) and equipped with a dual injector valve (Rheodyne Model 9725i PEEK injector, IDEX Health & Science LLC, CA, USA.^[^ ^85^ ^]^ To immobilize the mortalin substrate binding domain (SBD) or full‐length mortalin, self‐assembled monolayers (SAMs) of 3‐mercaptopropionic acid (10 × 10^−3^ m in ethanol) were formed by immersing the 16‐spot gold SPRi array chips for 2 h. The surface‐exposed carboxylic acid end groups of the SAMs were activated into amine‐reactive *N*‐succinimidyl esters by incubating them on a freshly prepared mixture of 0.35 m EDC and 0.1 m NHS solution for 10 min. Following this step, 20 × 10^−6^ m solutions of the mortalin SBD, or the full‐length mortalin, in 20 × 10^−3^ m Tris HCL buffer with 100 × 10^−3^ m NaCl and pH 7.4, were immobilized onto the surface. To reduce nonspecific binding, the protein‐immobilized gold‐SAM array spots were incubated for 20 min with methoxy polyethylene glycol, a blocking polymer (0.5% in pH 7.4 phosphate buffer). For the binding study, various SHetA2 concentrations were prepared in 1% dimethylformamide (DMF) in pH 7.4 phosphate buffer. Real‐time changes in percentage reflectivity (Δ%*R*) resulting from interactions between SHetA2 and either mortalin SBD or full‐length mortalin were recorded. In this method, the baseline signal does not increase unless there is a physical interaction between the added molecule and the immobilized molecule.^[^ ^85^ ^]^ To obtain binding curves and perform kinetic analysis as described in the previous report, real‐time binding of SHetA2 to either mortalin SBD or full‐length mortalin was monitored.^[^ ^36^ ^]^ Pre‐ and post‐binding images were obtained and analyzed using ImageJ software to obtain 3D representations of net changes.

### NMR Experiments

All NMR experiments were carried out on an Agilent INOVA 600 MHz spectrometer with a triple resonance probe. Sample temperature was regulated at 25 °C. NMR signals and chemical shifts, which were positions of the signals in the spectra, were assigned to backbone atoms based on the following three‐dimensional experimental datasets on a protein sample enriched with both ^13^C and ^15^N: NHSQC, HNCA, HNcoCA, HNCACB, HNcoCACB, HNCO and HNcaCO. Then, SHetA2 stock solution in DMSO was titrated to a ^15^N‐enriched protein sample at protein to ligand molar ratios of 1:0.2, 1:0.4, 1:0.6, 1:0.8, 1:1, 1:2, 1:4 and 1:8, a series of two‐dimensional NHSQC data were acquired. As controls, titration experiments were repeated for the same amounts of solvent but without SHetA2 to another ^15^N‐enriched sample. Chemical shift perturbations^[^ ^37^ ^]^ were obtained by measuring displacement of signals in a titration NHSQC spectrum relative to those in a corresponding control spectrum. Lowest energy docking conformations were derived using relation $\Delta G=RT\ln\;\frac{\mathrm{KD}}{1\;\mathrm{mol}\;L^{-1}}\;$ with gas constant *R* = 8.31 J K^−1^ mol^−1^ and *T* = 310 K.

### Molecular Docking

AutoDock 4.2^[^ ^86^ ^]^ was used for all the docking experiments and the results were analyzed using AutoDock Tool (ADT). The ligands were allowed to rotate freely though single bonds during the docking process. For protein, the crystal structure of mortalin substrate binding domain (SBD) (identification code 3N8E) was downloaded from Protein Data Bank. AutoGrid was run first to prepare the coordinates system and then the Lamarckian Genetic Algorithm was used with a population size of 150 and 25 million maximum evaluations for 10 runs for AutoDock.

### Cell Culture

Human ovarian cancer cells lines A2780, OV‐90, SKOV3, CAOV3, and HOSE were cultured in RPMI1640 medium containing 10% FBS and 1× antibiotic/antimycotic. OVCAR3 cells were extra supplemented with 10% FBS (final 20%) and 1% Insulin. HOSE cells and FT cells were cultured in collagen pre‐treated plates in DMEM‐high glucose medium with 10% FBS and 1× antibiotic/antimycotic. Cell lines were tested for mycoplasma contamination annually by PCR analysis by IDEXX Research. All experiments were performed with cell lines free of mycoplasma contamination. All experiments were performed in triplicate and repeated at least three times unless otherwise mentioned.

### Western Blotting

Whole cell protein extracts were obtained from cultures treated with either 10 × 10^−6^ m SHetA2 or DMSO for various durations using the M‐PER Mammalian Protein Extraction Reagent, as previously described.^[^ ^28^ ^]^ Cultures collected at the 0 time point, prior to treatment and cultures treated with the same amount of DMSO solvent used for the highest SHetA2 dose administered were used as negative controls. Protein concentrations were determined using the BCA reagent, and equivalent amounts of protein (20–35 µg) were separated by SDS‐PAGE, transferred onto a polyvinylidene difluoride (PVDF) membrane, and subjected to immunoblotting with specific primary antibodies for overnight at 4 degree, followed by appropriate HRP‐conjugated secondary antibodies for 2 h. Subsequently, membranes were washed and signals were developed using ECL reagents (BioRad). The same blots were subjected to stripping buffer treatment, followed by blocking with skimmed milk, and re‐probing with either anti‐GAPDH, anti‐α‐Tubulin, anti‐Tom20, or anti‐β‐actin antibodies for normalization purposes. Primary antibodies were utilized at dilutions recommended by the manufacturer, while secondary antibodies were used at a dilution of 1:4000–5000. Protein bands were visualized and quantified using a ChemiDoc imaging system with Image Lab Software (BioRad).

### Antibody Production

A polyclonal antibody to the mortalin MLS (Mort‐MLS) was generated under a contract to Pierce Biotechnology, Inc (Rockford, IL). A peptide consisted of a 19 aa sequence of the mortalin MLS predicted to be antigenic was synthesized and conjugated with keyhole limpet hemocyanin (KLH). The rabbit was pre‐bled and injected with the peptide on Day 0. Boost injections with the peptide were administered on days 14, 42, and 56 after the initial injection. The rabbit was bled for production of polyclonal antibody on days 28, 56, 70, and 72. All blood specimens were shipped to the University of Oklahoma on Day 77.

### Mitochondrial and Cytoplasmic Subcellular Fraction Enrichment

Cytoplasmic and mitochondrial subcellular protein fractions were isolated from A2780, SKOV3, and OV90 ovarian cancer cells. The cells were seeded and treated with or without 10 × 10^−6^ m SHetA2 for 24 h. Approximately 5 × 10^7^ cells were subjected to subcellular fractionation using the Mitochondria/Cytosol Fractionation Kit following the manufacturer's protocol.

### Immunocytochemical Staining

FT cultures treated with 10 × 10^−6^ m SHetA2 or the same volume of DMSO for 24 h were released from culture plates by trypsin, rinsed in PBS and pelleted by centrifugation. The pellets were fixed in formalin, embedded in paraffin and sectioned. The sections were stained with the mortalin‐MLS antibody diluted 1:1000 and secondary antibody poly‐HRP‐IgG using an automated (Leica Bond III) IHC work station. The reagents were purchased from Leica Biosystems, Nuccloch, Germany. The product numbers for the reagents were: retrieval solution, AR9961(H1); peroxidase blocking reagent, Ig‐G linker, and HRP‐IgG are in one kit, DS9800. The primary antibodies were: mortalin‐MLS custom‐made as described above; Anti‐Grp75/MOT, Abcam cat# 53098. Briefly, formalin fixed paraffin embedded cell pellets sectioned at desired thickness (4–8µm) and mounted on positively charged slides. The slides were dried overnight at room temperature and incubated at 60 °C for 45 min followed by deparaffinization and rehydration in an automated Multistainer (Leica ST5020). Subsequently, these slides were transferred to the Leica Bond‐III, treated for target retrieval at 100 °C for 20 min in a retrieval solution, either at pH 6.0 or 9.0. Endogenous peroxidase was blocked using peroxidase‐blocking reagent, followed by the selected primary antibody incubation for 60 min. For the secondary antibody, post‐primary IgG‐linker and/or Poly‐HRP IgG reagents was used. Detection was done using 3,3′‐diaminobenzidine tetrahydrochloride (DAB), as chromogen and counterstained with hematoxylin. Completed slides were dehydrated (Leica ST5020), and mounted (Leica MM24). Appropriate negative controls (omission of primary antibody) were parallel stained.

### Mitochondria Metabolism

Various parameters of mitochondrial respiration were assessed using the 96‐well XF analyzer in conjunction with the XF Cell Mito Stress Test Kit (Seahorse Bioscience, Lexington, MA, USA). The assay was conducted according to the manufacturer's protocol. 12 h before the assay, XF sensor cartridges were hydrated with the provided calibrant and then incubated at 37 °C without CO_2_ to maintain a pH of 7.4 at 37 °C. Approximately 3 × 10^4^ cells were seeded in the 96‐well XF assay plate, both with and without 10 × 10^−6^ m SHetA2, in unbuffered XF assay medium. 30 min prior to the assay, the plates were washed with the assay medium and then incubated for 1 h at 37 °C without CO_2_. Oligomycin (2 × 10^−6^ m), carbonyl cyanide p‐trifluoro‐methoxyphenyl hydrazone (FCCP; 500 × 10^−9^ m), and a mixture of antimycin A and rotenone (1 × 10^−6^ m each) were added into the appropriate ports of the cartridge and calibrated within the instrument. Upon calibration completion, the cell culture plate was loaded into the instrument, and the assay was performed according to the standard template, allowing for the measurement of the OCR.

### PLA

Ovarian cancer cell lines underwent a PLA to validate the interaction between IP3R and mortalin in SHetA2‐treated cells, following the manufacturer's guidelines. Cells were fixed and permeabilized with methanol/PBS and PBST, respectively. After blocking, primary antibodies (Mortalin and IP3R at 1:100 dilution) were applied and washed. PLUS and MINUS PLA probes were added and incubated, followed by ligation and amplification steps. Slides were washed and mounted with Duolink In Situ Mounting Medium with DAPI for imaging using a confocal microscope Leica SP2 (Leica, Wetzlar, Germany) at 63×. Data was analyzed based on PLA foci/cells.

### Coimmunoprecipitation Assay

To investigate the interaction between IP3R and Mortalin proteins, a co‐immunoprecipitation assay was conducted with OV90 cells using the Pierce Co‐Immunoprecipitation Kit, followed by immunoblotting. A total of 10µg of IP3R and Mortalin antibodies were utilized for coupling to agarose beads following the manufacturer's protocol. The antibody‐coupled beads were then incubated with 500 µg of protein lysate overnight at 4 °C, followed by three washes with the buffer provided in the kit. The immuno‐precipitated complexes were collected according to the protocol instructions. Subsequently, the immuno‐precipitates were resuspended in sample buffer and heated for 5 min at 95 °C. Equal volumes of the immuno‐precipitated proteins were separated by 4–15% gradient SDS‐PAGE and transferred onto a PVDF membrane. Both the total cell proteins and the immuno‐precipitated proteins from the Co‐IP of IP3R, VDAC1, and Mortalin were probed with anti‐IP3R and Mortalin antibodies. The bands were then detected and analyzed using Image Lab 5.2.1 software (Bio‐Rad).

### Mitochondrial Ca^2^⁺ Level Detection

To evaluate mitochondrial Ca^2^⁺ levels following SHetA2 exposure, Rhod‐2 AM (10 × 10^−6^ m, 30 min) was used as a fluorescent indicator. Ovarian cancer and normal cells were seeded at densities of 5 × 10⁴ cells per well in six‐well plates for flow cytometry (FACS) analysis and 5 × 10^3^ cells per well in 96‐well plates for plate reader analysis. Cells were treated with 10 × 10^−6^ m SHetA2 or the same volume of DMSO vehicle for 24 h, followed by incubation with 10 × 10^−6^ m Rhod‐2 AM for 30 min. After three washes with PBS, fluorescence was detected using (1) flow cytometry (FACS) and (2) a plate reader, with excitation/emission wavelengths set at 552 nm/581 nm.

### Ca^2+^ Localization Assay

To investigate the subcellular localization of Ca^2+^ in cells following exposure to SHetA2, MitoView Green was used to stain mitochondria, while Rhod‐2AM (10 × 10^−6^ m for 30 min) was employed to detect mitochondrial Ca^2+^ concentration. Approximately 5 × 10^4^ ovarian cancer and normal cells were seeded in chamber slides purchased from Lab‐Tek II. Live cells were incubated with Rhod‐2AM (10 × 10^−6^ m for 30 min) followed by MitoView Green (200 × 10^−6^ m for 30 min) incubation. The cells were then washed thrice with PBS and imaged using a confocal microscope (Leica SP8, Leica) with excitation/emission wavelengths of 552 nm/581 nm for Rhod‐2AM and 490 nm/523 nm for MitoView Green, at 63× magnification.

### Immunofluorescence

Chamber slides from Lab‐Tek II, were seeded with 1 × 10^4^ ovarian cancer cells in 1 ml medium and incubated up to 50–70% confluence. Cells were treated with SHetA2 or DMSO for 24 h. Following treatment, the cells were stained with MitoTracker Red CMXRos‐M7512 for 30 min in medium, washed twice with PBS, and then fixed with 4% paraformaldehyde (pH 7.4) for 30 min at room temperature. After fixation, the cells were washed three times with PBS and permeabilized with 0.5% Triton X‐100 in PBS (pH 7.4) for 5 min. Following three PBS washes, the cells were incubated in PBS containing 0.1% BSA for 30 min at room temperature, and then with primary antibodies for 2 h at room temperature at a 1:200 dilution. After the incubation period, the cells were washed three times with PBS and then incubated with secondary antibodies tagged with fluorochromes (Alexa Fluor 488 or 647) at a 1:500 dilution for 1 h at room temperature. Following three additional washes with PBS, the cells were mounted using Prolong Gold mounting medium with DAPI and 1 mm thick cover glass. The mounted cells were left to dry overnight at room temperature and then imaged using a confocal microscope (Leica SP2, Leica) at 63× magnification.

### Detection of Mitophagy

For the quantitative analysis of mitophagy, approximately 5 × 10^3^ cells were seeded in triplicate in black clear bottom Cell Carrier ‐96 well plates, with or without 5 or 10 × 10^−6^ m SHetA2, for 24, 36, and 72 h. The Mitophagy Detection Kit was employed to quantify mitophagy, following the manufacturer's recommendations. Briefly, cells were washed twice with RPMI and then incubated with 100 nmol of Mitophagy Dye (working solution) diluted in RPMI at 37 °C for 30 min. After this incubation, cells were washed twice with RPMI and complete RPMI media (RPMI with 10% FBS and 1× antibiotic/antimycotic) was added, followed by treatment with 10 × 10^−6^ m SHetA2 or an equivalent volume of DMSO for 24 h. To observe the colocalization of the Mitophagy Dye and lysosomes, 1 µmol L^−1^ of Lyso Dye (working solution) diluted in RPMI was incubated at 37 °C for 30 min. Nuclei were stained with 100 × 10^−9^ m Hoechst to quantify the total number of cells. Mitophagy quantification was performed by dividing the colocalized spots (observed by Mitophagy Dye and Lysosome Dye) by the total number of mitochondria (total spots by Mitophagy Dye) per cell. Images were captured using the Operetta CLS high‐content analysis system (Perkin Elmer) with excitation/emission wavelengths of 561 nm/650 nm for Mitophagy Dye, 488 nm/502–554 nm for Lysosome Dye, and 361 nm/497 nm for Hoechst. Image analysis was conducted using Columbus Harmony 4.5 software.

### Image Analysis of Mitophagy

To visualize the colocalization of mitochondria with lysosomes in ovarian cancer cells, MitoTracker Red was utilized to label mitochondria, while Lyso Dye from the Mitophagy Detection Kit was employed to identify lysosomes. Approximately 5 × 10^4^ A2780 and SKOV3 cells were seeded in Lab‐Tek II chamber slides. The cells were then treated either with 10 × 10^−6^ m SHetA2 or an equivalent volume of DMSO for 24 h. To observe the colocalization of mitochondria and lysosomes, MitoTracker Red (1 × 10^−6^ m) and 1 µmol L^−1^ of Lyso Dye (working solution) were diluted in RPMI and then incubated at 37 °C for 30 min. Following this incubation, the supernatant was discarded, and the cells were washed twice with RPMI. Subsequently, they were covered in Prolong Gold mounting medium with DAPI and left to dry overnight at room temperature. The samples were then imaged using a confocal microscope (Leica SP2, Leica) with excitation/emission wavelengths of 561 nm/650 nm for MitoTracker Red and 488 nm/502‐554 nm for Lyso Dye, at 63× magnification.

### Mitochondrial Morphology Analysis

To evaluate mitochondrial morphology, live cells were stained with MitoView Green (200 × 10^−6^ m) for 30 min, followed by live cell confocal microscopy while maintaining conditions at 37 °C and 5% CO_2_. Approximately 5 × 10^4^ cells were plated on chamber slides with either SHetA2 or DMSO, followed by nuclei staining with 100 × 10^−9^ m Hoechst for 10 min at room temperature. The cells were subsequently washed six times with PBS. Images were captured using a confocal microscope (Leica SP2, Leica) at 63× magnification and zoomed up to 3× or 4× to obtain a single cell per image for accurate image analysis. Mitochondrial networks were analyzed using a modified method, as described by Valente et al., and Rai et al.,^[^ ^30^, ^87^ ^]^ utilizing the FIJI distribution by ImageJ and an open‐source macro tool called Mitochondrial Network Analysis (MiNA), designed by the authors. In summary, images were split into their red‐green‐blue color channels, with only the mitochondria channel being analyzed. The images were processed using the mean filter before being processed by the macro. The macro was executed with default settings, except processing by the median filter was disabled. Individual and network mitochondria were counted, and the network's branching and branch length were measured. Individual mitochondria were defined as segments with no branches, while network mitochondria were defined as segments with at least one branching arm.

### Coimmunoprecipitation Assay for Mass Spectrometry LC‐MS/MS Analysis

To investigate the interactions between mortalin and its client proteins, ovarian cancer cell lines (OV90 and A2780) and primary fallopian tube (FT) cultures (FT007 and FT032) were treated with SHetA2 or vehicle control for 4 h at 10 × 10^−6^ m. Protein extracts were then collected, and coimmunoprecipitation was performed with Mortalin antibody by using the Pierce Co‐Immunoprecipitation Kit. In brief, immunoprecipitation, approximately 500 µg of protein lysate was incubated overnight at 4 °C with agarose beads pre‐coupled to 10 µg of mortalin antibodies, following the manufacturer's protocol. The obtained samples lysate were then submitted to the core laboratory facility at Harvard for mass spectrometry analysis.as previously described.^[^ ^88^ ^]^

### Electron Microscopy

Cultures treated with 10 × 10^−6^ m SHetA2 or DMSO solvent for 17 h were fixed (4% Paraformaldehyde, 2% Gluteraldehyde in 0.1 m sodium cacodylate) on ice for 5 h followed by rinsing in 0.1 m sodium cacodylate, post fixation in 1% osmium tetroxide for 1.5 h, rinsing in 0.1 m sodium cacodylate and incubation in 2% paraformaldehyde at 4 °C overnight. The next day, the cells were dehydrated through a graded series of ethanol solutions, infiltrated with resin (6.2 g Epon, 4.4 g Araldite, 12.2 g DDSA)/ethanol series (3:1, 1:1, 1:3) and then embedded and cured for 24 h. Ultrathin sections were placed on glow discharged, 400 mesh copper grids and stained in Sato's Lead. Images were obtained using a Hitachi H7600.

### Transfection of DNA Plasmids into Cell Cultures

A2780, SKOV3, or HOSE cells were grown to 70–80% confluency in a 6‐well dish. Complete media was removed and serum free media was added immediately before transfection. A total of 2–4 µg DNA (GFP‐LC3‐tdTomato,) was added to 100 µL serum‐free media with a 3:1 ratio of Xtreme Gene (Roche) transfection reagent.  The mixture was allowed to incubate at room temperature for 30 min and then dropwise added to plated cells in serum free media.  Media was changed immediately the next morning to complete media and cells remained for at least 8 h in complete media before treatment with 10 × 10^−6^ m of SHetA2 or DMSO for multiple time points.

The A2780 cells was transfected with Bcl‐2 (pCDH‐puro‐Bcl‐2) or Bcl‐XL (3120 pSFFV‐neo BCL‐XL) plasmids by using the lipofctamine‐3000 transfection reagent. Approximately 5 × 10^3^ cells were seeded in Opti‐MEM media along with the transfection mixture following the manufacturer's instructions. Following 24 h of transfection, the media was replaced with RPMI1640 medium supplemented with 10% FBS and 1× antibiotic/antimycotic, and the cells were further incubated at 37 °C for an additional 24 h. Subsequently, after a total of 48 h post‐transfection, 10 × 10^−6^ m of SHetA2 or DMSO was added to the cells for another 24 h. The cells were then collected, washed with PBS, and analyzed for western blotting.

### Fluorescence Imaging of LC3‐tdTomato

SKOV3, A2780, or HOSE cells transiently transfected with GFP‐LC3‐tdTomato construct using XtremeGene HP (Roche Applied Bioscience, Indianapolis, IN, USA) according to the manufacturer's instructions were treated with SHetA2 added at various time points after transfection and imaged with 555 nm/28 nm excitation and 617/73 emission filters using a Nikon TE2000‐E at 40× magnification.

### Detection of Autophagic Vesicles by Acridine Orange

SKOV3 or HOSE cells were seeded in 6‐well plates and allowed to grow to 70–80% confluency overnight. Cells were then left untreated, treated with 100 nmol L^−1^ rapamycin for 48 h as a positive control for autophagy induction, or with 10 × 10^−6^ m SHetA2 in DMSO for various time points. At the appropriate time points, cells were incubated with 1 µg mL^−1^ acridine orange (MilliporeSigma, Burlingon, MA, USA) in the media for 15 min at 37 °C. Then the wells were washed and imaged with an inverted microscope.

### Coimmunoprecipitation Assay for Mass Spectrometry LC‐MS/MS Analysis

To investigate the interactions between mortalin and its client proteins, ovarian cancer cell lines (OV90 and A2780) and FT cultures (FT007 and FT032) were treated with SHetA2 or vehicle control for 4 h at 10 × 10^−6^ m. Protein extracts were then collected, and coimmunoprecipitation was performed with the D13H4 Mortalin antibody using the Pierce Co‐Immunoprecipitation Kit. In brief, immunoprecipitation, approximately 500 µg of protein lysate was incubated overnight at 4 °C with agarose beads pre‐coupled to 10 µg of mortalin antibodies, following the manufacturer's protocol. The obtained samples lysate were then submitted to the core facility for mass spectrometry analysis.

### Methods for Protein Sequence Analysis by Mass Spectrometry

Mass spectrometry analysis was performed at the Taplin Biological Mass Spectrometry Facility (Harvard Medical School, Boston, MA). Proteins were digested with a solution of 50 × 10^−3^ m ammonium bicarbonate solution containing 12.5 ng µL^−1^ modified sequencing‐grade trypsin (Promega) at 4 °C. After 45 min the excess trypsin solution was removed and replaced with 50 × 10^−3^ m ammonium bicarbonate solution. Samples were then placed in a 37 °C room overnight. Peptides were later extracted by removing the ammonium bicarbonate solution, followed by one wash with a solution containing 50% acetonitrile and 1% formic acid. The extracts were then dried in a speed‐vac (≈1 h). The samples were then stored at 4 °C until analysis. On the day of analysis the samples were reconstituted in 5–10 µL of HPLC solvent A (2.5% acetonitrile, 0.1% formic acid). A nanoscale reverse‐phase HPLC capillary column was created by packing 2.6 µm C18 spherical silica beads into a fused silica capillary (100 µm inner diameter × ≈30 cm length) with a flame‐drawn tip. After equilibrating the column each sample was loaded via a Famos auto sampler (LC Packings, San Francisco CA) onto the column. A gradient was formed and peptides were eluted with increasing concentrations of solvent B (97.5% acetonitrile, 0.1% formic acid). As peptides eluted they were subjected to electrospray ionization and then entered into an LTQ Orbitrap Velos Pro ion‐trap mass spectrometer (Thermo Fisher Scientific). Peptides were detected, isolated, and fragmented to produce a tandem mass spectrum of specific fragment ions for each peptide. Peptide sequences (and hence protein identity) were determined by matching protein databases with the acquired fragmentation pattern by the software program, Sequest (Thermo Fisher Scientific). All databases include a reversed version of all the sequences and the data was filtered to between a one and two percent peptide false discovery rate.

### siRNA and Apoptosis Assay

Scrambled or Beclin1 siRNA was transfected using the HiPerfect transfection reagent. Approximately 5 × 10^3^ cells were seeded in Opti‐MEM media along with the transfection mixture following the manufacturer's instructions. Following 24 h of transfection, the media was replaced with RPMI1640 medium supplemented with 10% FBS and 1× antibiotic/antimycotic, and the cells were further incubated at 37 °C for an additional 24 h. Subsequently, after a total of 48 h post‐transfection, 10 × 10^−6^ m of SHetA2 or DMSO was added to the cells for another 24 h. The cells were then collected, washed with PBS, and analyzed for western blotting or apoptosis assay. The apoptosis assay was performed using the Alexa Fluor 488 annexin V/Dead Cell apoptosis kit (Invitrogen) according to the manufacturer's protocol and assessed using flow cytometry.

### MTT‐Cytotoxicity Assay and Drug Interaction Analysis

Cell viability following drug treatments was assessed using the CellTiter 96 Non‐Radioactive Cell Proliferation Assay (MTT). Cells were seeded into 96‐well plates and allowed to incubate overnight. The next day, cells were treated with various drugs, including SHetA2, DMSO, or CQ, according to the experimental design, for durations of 24 and 72 h. The autophagy inhibitor CQ (50 × 10^−6^ m) was supplemented with or without SHetA2 treatment. Following the respective incubation periods, the MTT assay was conducted by adding 15 µL of MTT dye to each well and incubating for 1 h. Subsequently, 100 µL of stop/solubilization solution was added to each well and left overnight in a humidified 37 °C, 5% CO_2_ incubator. The absorbance of the MTT dye at 570 nm and background wavelengths at 620 nm were measured using a BioTeK SYNERGY H1 spectrophotometer (BioTeK, Winooski, VT, USA). The optical densities (ODs) of the individual SHetA2 treated wells were normalized to the average ODs of DMSO treated control wells, while the individual ODs of the wells treated with the combination of SHetA2 and CQ were normalized to the average ODs of CQ treated wells.

### OV90 Xenograft Survival Study

The animal study was conducted following approval by the Institutional Animal Care and Use Committee (Protocol #21‐052‐CHI) at the University of Oklahoma Health Sciences Center, Oklahoma City, OK, USA and in accordance with standard animal protocols. Female 5‐week‐old SCID mice were obtained from Envigo Sales, Indianapolis, IN, USA and given a 72‐h acclimation period. Subsequently, the mice were subcutaneously injected with 1 × 10^6^ OV90 cells suspended in 100 µL sterile normal saline. Tumor size was monitored three times per week using calipers and the formula ((width^2^ × length)/2). Upon reaching an average volume of 100 mm^3^, the mice were randomized into two groups of 10 mice each, ensuring no significant differences in tumor size between the groups at the start of treatment (ANOVA, *p* > 0.05). Treatment commenced with two groups receiving either control placebo (30% Kolliphor HS 15 in water, administered daily) or 60 mg kg^−1^ SHetA2 daily via oral gavage. Animals body weights were measured every other day during the treatment days, and their health was monitored daily. After 11 days of treatment, all mice were euthanized by controlled CO_2_ inhalation, followed by cardiac puncture to ensure death. Tumors were collected at necropsy, with a portion of each tumor fixed in paraformaldehyde and embedded in paraffin, while another portion was snap‐frozen in liquid nitrogen.

### A2780 Xenograft Survival Study

The animal study was conducted following approval by the Institutional Animal Care and Use Committee (Protocol #05060). Eight week old female BALB/c/*nu/nu* mice were purchased from the Jackson Laboratory (Farmington, CN, USA) and given a 1 week acclimation period. Thirty mice were injected with 5 × 10^6^ A2780 cells suspended in 100 µL sterile normal saline subcutaneously into the right flank. The mice were monitored three times per week for tumor establishment.  The tumor volumes were measured with calipers.  The length, width, and height of each tumor was recorded and the volumes calculated from the following formula: Volume = (length × width^2^ × π)/2. Ten days later, treatment with SHetA2 was initiated at 60 mg kg^−1^ suspended in PEG400 administered by oral gavage three times per week (M, W, F). Twenty four days after tumor cell injection, the tumors were removed. The 16 mice that had peritoneal invasion were euthanized. The 14 mice that did not have peritoneal invasion were considered to be completely resected by an experienced surgeon (T.L.), sutured, kept warm, given antibiotics and carefully monitored post‐surgery. Thirty‐one days after tumor cell injection/1‐week post‐surgery the mice were divided into two groups. One group was gavaged with 60 mg kg^−1^ SHetA2 in suspended PEG400 and the other with PEG400 placebo three times per week (M, W, F). During the remainder of the experiment, the mice were monitored daily by veterinary personnel and euthanized based on humane endpoints. One‐hundred and nine days after tumor cell injection, all remaining mice were terminated.

### Statistical Analysis

A one‐way ANOVA was used for comparison of multiple groups and a two‐way ANOVA was use for comparisons involving multiple doses and treatment times. For the mortalin interactome results evaluated by mass spectrometry analysis, peak intensities were normalized to the lowest peak intensity value for each protein. Outliers that were greater than threefold different from the matching two replicates were removed from the analysis. Proteins identified to have been released from mortalin by SHetA2 must have had significant differences by a two‐way ANOVA with Tukey's multiple comparisons test showing: 1) the SHetA2‐treated group having less peak intensity compared to the control group and 2) the control group having less normalized peak intensity compared to the negative control group. For the OV90 animal model, the repeated measures ANOVA was used with Dunnett's multiple comparisons test for tumor volume and body weight measurements. Linear regression analysis was performed to determine the 95% confidence intervals of the slopes of the lines to compare mouse body growth rates between groups during the treatment periods. The nonparametric Kruskal–Wallis test with Dunnett's multiple comparisons test was used to compare final tumor weights and organ‐to‐body weight ratios. For the A2780 survival study, the Gehan–Breslow–Wilcoxen test was done. The *p* values for primary analysis and adjusted *p* values for multiple comparisons of <0.05 were considered statistically significant. All analyses were conducted using Graph Pad Prism 10.0 software.

## Conflict of Interest

The authors declare no conflict of interest.

## Author Contributions

V.C. and D.M.B. contributed to conceptualization and writing original the original draft. V.C., J.G., D.Z., S.K., T.L., and D.M.B contributed to methodology. V.C., J.G., R.R. C.W., A.T.L., T.L., and D.M.B. contributed to the investigation and validation. V.C., J.G., R.R., D.Z., C.W., S.K., A.T.L., T.L., L.A., and D.M.B. contributed to formal analysis and visualization. D.M.B. contributed to resources, project administration, and funding acquisition. V.C., D.Z., S.K., and D.M.B. contributed to supervision. All authors read and edited original draft.

## Ethics Approval and Consent to Participate

The fallopian tube primary cultures were grown from surgical specimens obtained from patients giving informed consent under the University of Oklahoma Health Sciences Center (OUHSC) Institutional Review Board (IRB) protocol #5563. The mouse xenograft experiments were performed in compliance with the Animal Welfare Act under approval by the OUHSC Institutional Animal Care and Use Committee (IACUC) of protocols 05060 and 21‐052‐CHI.

## Consent for Publication

All authors have approved this final version of the manuscript.

## Supporting information



Supporting Information

## Data Availability

The datasets used and/or analyzed during the current study are available from the corresponding author on reasonable request.
