# Supplementary material for: Mortalin and PINK1/Parkin‐Mediated Mitophagy Represent Ovarian Cancer‐Selective Targets for Drug Development
Source: Adv Sci (Weinh). 2025 Jul 20;12(37):e05592. doi: 10.1002/advs.202505592 (PMC12499392; doi:10.1002/advs.202505592)
Supplement: Supplementary file 1 — Supporting Information [file ADVS-12-e05592-s001.docx]

**Supplementary Figure-S1**


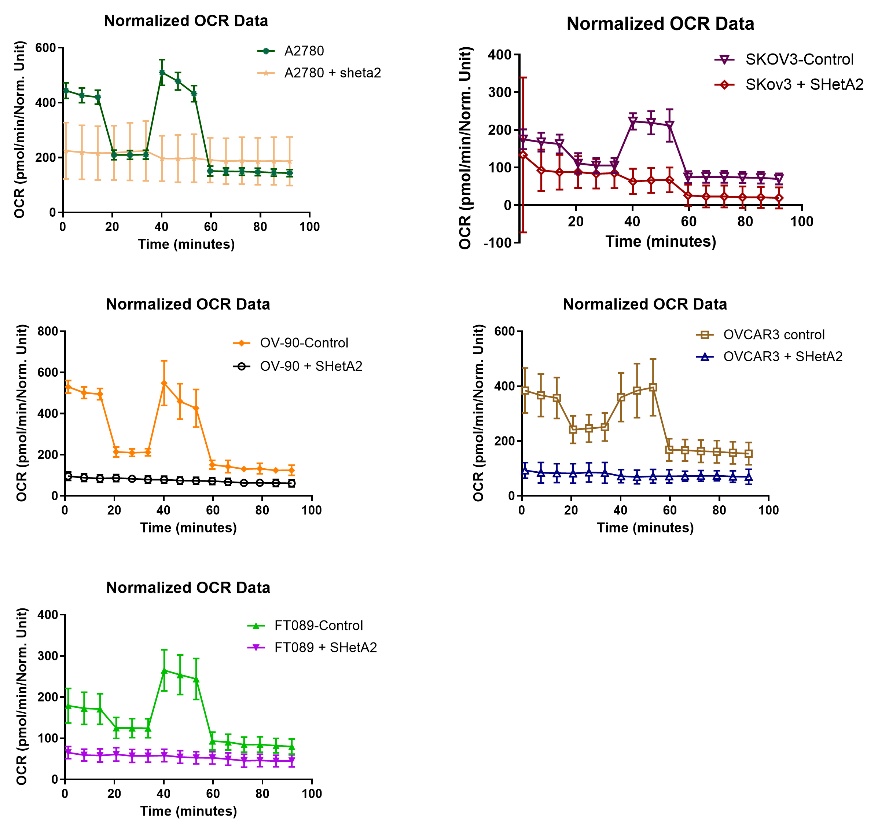


**Supplementary Fig. S1.** Mitochondrial metabolism is inhibited in cancer and non-cancer cells by SHetA2: Normal or ovarian cancer cells were treated with DMSO or 10 μM SHetA2 for 24 hrs. and OCR was measured using the Seahorse XFe96 analyzer and cell mito-stress assay.

**Supplementary Figure-S2**


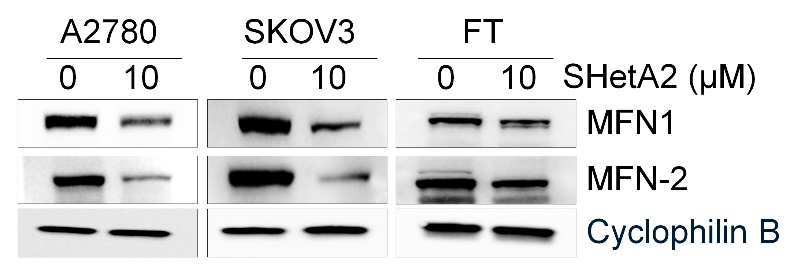


**Supplemental Fig. S2.** Molecular mechanism of SHetA2-induced regulation of mitochondrial fusion and fission proteins: A2780 and SKOV3 ovarian cancer cell lines, along with non-cancerous fallopian tube (FT) cells, were treated with either DMSO or 10 µM SHetA2 for 24 hours. Cells were then analyzed by immunoblotting for expression of mitochondrial fusion markers MFN1 and MFN2. Cyclophilin B was used as a loading control.

**Supplementary Figure-S3**


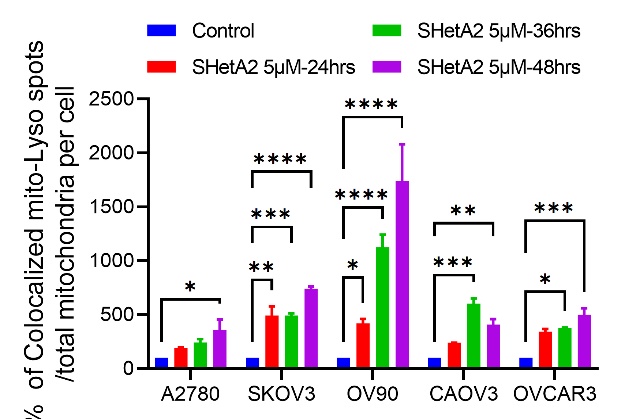


**Supplementary Fig. S3:** Cellular response to SHetA2-induced mitochondrial damage and mitochondria-selective autophagy (mitophagy): Ovarian cancer cells treated with DMSO or 5µM SHetA2 for indicated time points were stained with Lyso Dye (green, recognizes Lysosomes) and Mito-Dye (red, recognizes mitochondria) and imaged by Operetta CLS™ high-content analysis system at 10X for bigger field. Mitophagy induction was quantified by dividing yellow spots by total mitochondria per cells and shown as bar graph. Data are shown as mean ± SD. “p” values are indicated  * p ≤ 0.05, ** p ≤ 0.01, *** p ≤ 0.001, **** p ≤ 0.0001 when compared with respective control.

**Supplementary Figure-S4**


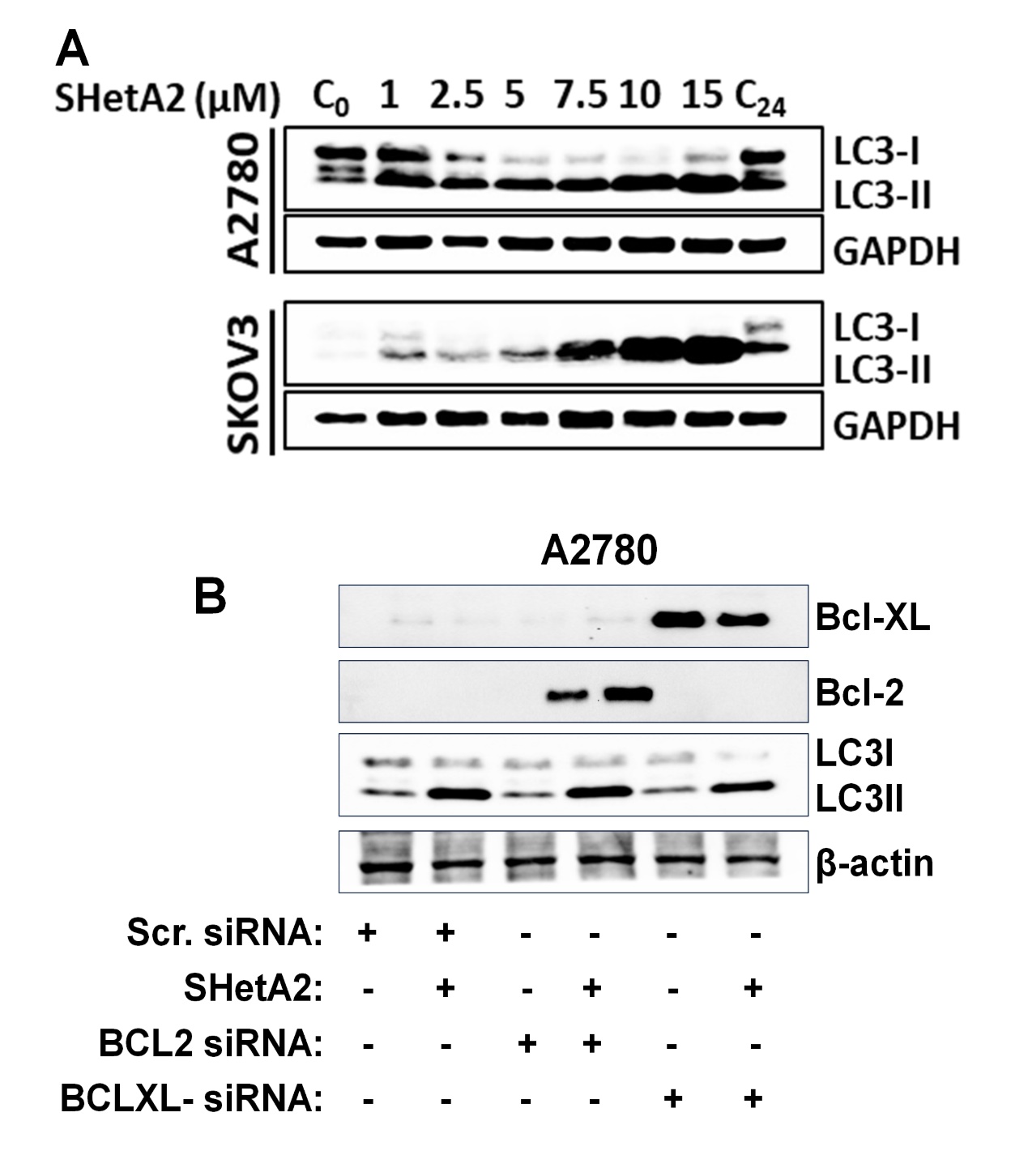


**Supplemental Fig. S4.**  Molecular mechanism of SHetA2-induced mitophagy: A) A2780 and SKOV3 ovarian cancer cell lines treated with DMSO or different concentration of SHetA2 for 24 hrs. were analyzed for LC3-I/II expression by immunoblotting. GAPDH was used as loading control. B) A2780 cells transiently transfected with Bcl2 and Bcl-XL vector (for their overexpression) or control vector were treated with DMSO or 10µM SHetA2 and the protein isolates were immunoblotted for autophagy marker LC3-I/II. The overexpression of transfected gene Bcl2 and Bcl-XL were confirmed by anti -Bcl2 and -Bcl-XL antibody respectively. β-actin was used as loading control

**Supplementary Figure-S5**


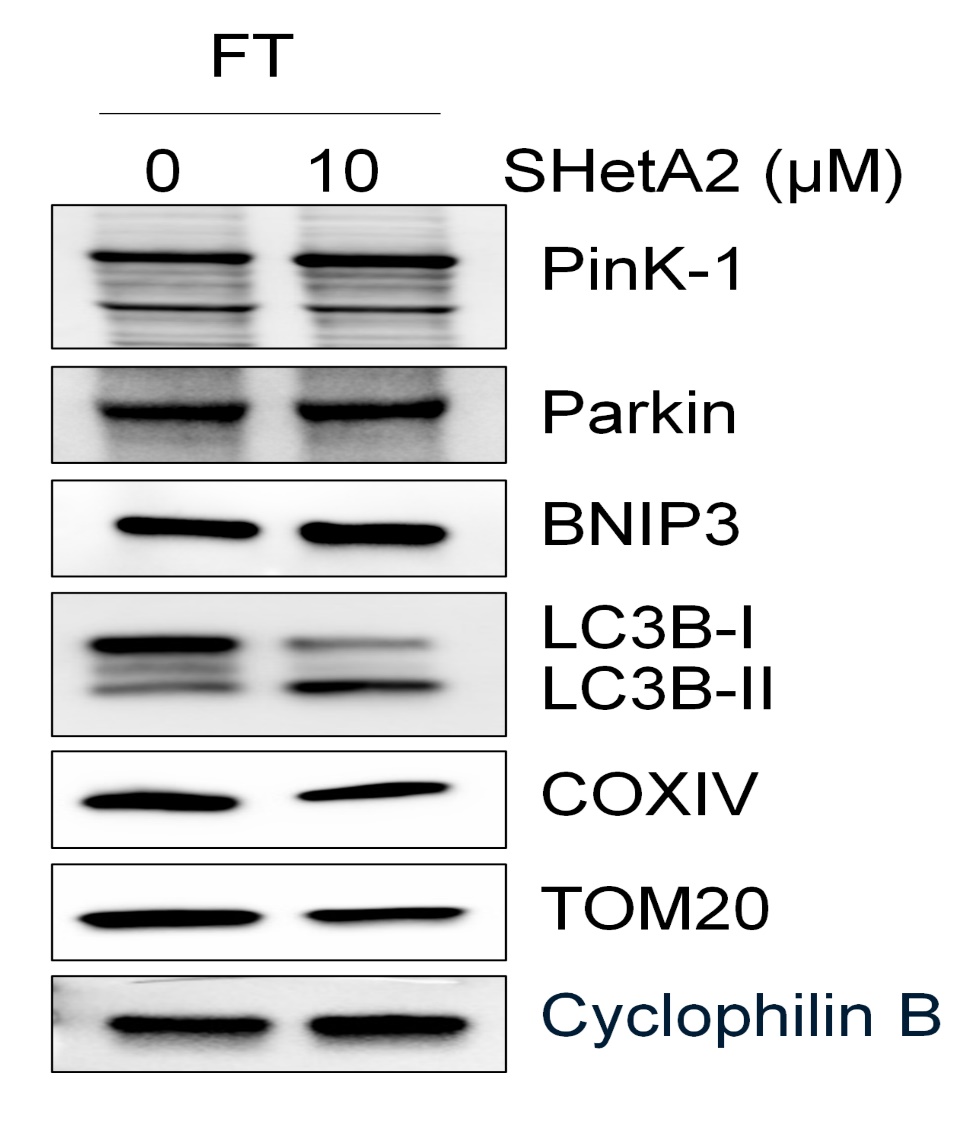


**Supplemental Fig. S5.** Molecular mechanism of SHetA2-induced mitophagy: Non-cancerous fallopian tube (FT) cells, were treated with either DMSO or 10 µM SHetA2 for 24 hours. Cells were then analyzed by immunoblotting for expression of mitophagy markers. Cyclophilin B was used as a loading control.
